# Synthesis of Metal–Organic Cages via Orthogonal Bond Cleavage in 3D Metal–Organic Frameworks

**DOI:** 10.1021/jacs.4c09431

**Published:** 2024-09-23

**Authors:** Sara Ruiz-Relaño, Dongsik Nam, Jorge Albalad, Alba Cortés-Martínez, Judith Juanhuix, Inhar Imaz, Daniel Maspoch

**Affiliations:** † Catalan Institute of Nanoscience and Nanotechnology (ICN2), CSIC and Barcelona Institute of Science and Technology, Campus UAB, Bellaterra, 08193 Barcelona, Spain; ‡ Departament de Química, Facultat de Ciències, Universitat Autònoma de Barcelona, Campus UAB, Bellaterra, 08193 Barcelona, Spain; § Alba Synchrotron Light Facility, Cerdanyola del Vallès, 08290 Barcelona, Spain; ∥ ICREA, Passeig Lluis Companys 23, 08010 Barcelona, Spain

## Abstract

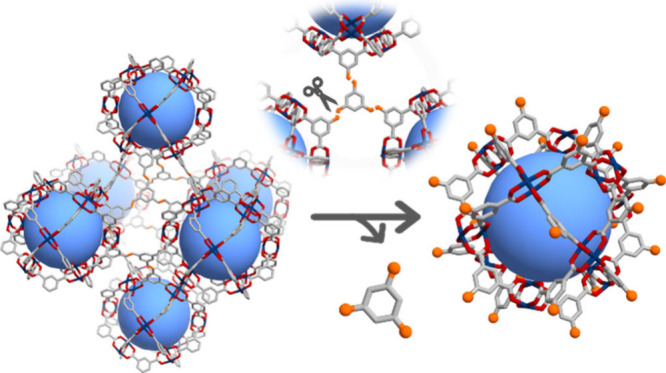

Herein we address the question of whether a supramolecular finite metal–organic structure such as a cage or metal–organic polyhedron (MOP) can be synthesized via controlled cleavage of a three-dimensional (3D) metal–organic structure. To demonstrate this, we report the synthesis of a Cu(II)-based cuboctahedral MOP through orthogonal olefinic bond cleavage of the cavities of a 3D, Cu(II)-based, metal–organic framework (MOF). Additionally, we demonstrate that controlling the ozonolysis conditions used for the cleavage enables Clip-off Chemistry synthesis of two cuboctahedral MOPs that differ by their external functionalization: one in which all 24 external groups represent a mixture of aldehydes, carboxylic acids, acetals and esters, and one in which all are aldehydes.

Bond formation and bond breaking are fundamental chemical processes. Although bond formation has traditionally driven most chemical syntheses and technologies,^1−6^ control of bond breaking at the molecular scale begun to garner interest due to its growing importance in emerging technologies and chemical strategies.^7−13^ For example, controlling bond cleavage in organic polymers has become essential for improving their recyclability and for developing new closed-loop recycling processes.^14−17^ Similarly, controlling bioorthogonal cleavage reactions has become crucial for liberating and activating prodrugs,^18^ reactivating proteins,^19^ and releasing bioconjugates.^20^ In this context, we recently introduced the concept of Clip-off Chemistry, whereby we design and synthesize novel molecules and materials with well-defined structures through orthogonal bond cleavage within molecular structures.^21−23^

Among the various types of molecular structures, reticular materials stand out as particularly appealing precursors for Clip-off Chemistry.^24,25^ Reticular materials can be viewed as the linkage of repetitive units or fragments that form when basic inorganic and/or organic building blocks are connected.^26−32^ These units or fragments, which can include clusters, cages, macrocycles, chains and layers, among others, can exhibit new properties and functions on their own. Therefore, they hold promise as a new source of molecules or materials if isolated from the reticular precursor. We devised Clip-off Chemistry to isolate these units or fragments via cleavage of the bonds (*e*.*g*. olefinic bonds by ozonolysis)^33^ that link them within reticular materials.

Herein, we report the first example of Clip-off Chemistry being applied to three-dimensional (3D) metal–organic frameworks (MOFs) to synthesize 0D metal–organic cages or polyhedra (MOPs).^29,34−42^ This work entails the quantitative orthogonal bond cleavage within a 3D structure, followed by isolation and characterization of the unconnected, “released” MOPs (Figure 1).

**Figure 1:**
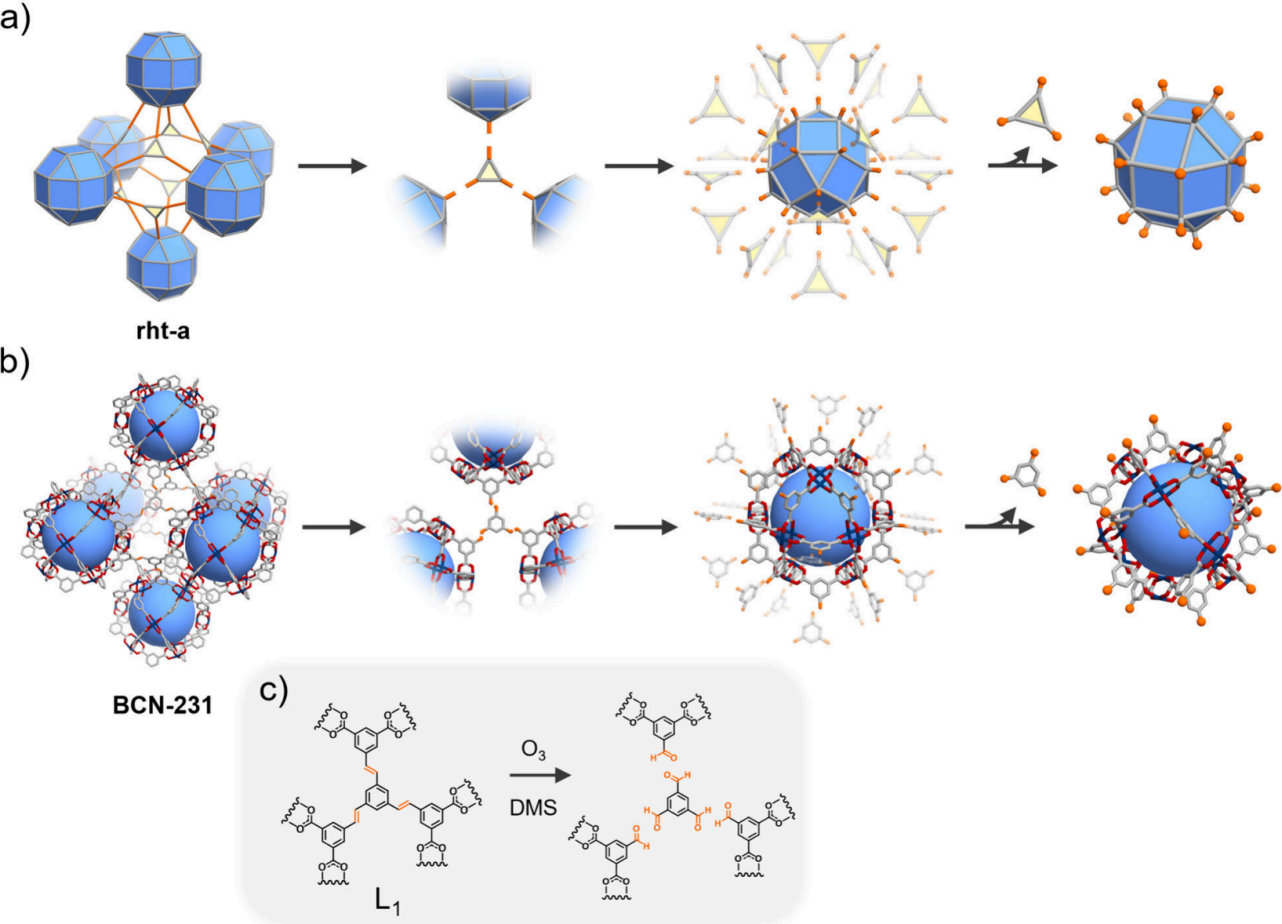
(a) Schematic of the synthesis and isolation of a cuboctahedral metal–organic polyhedron (MOP) via orthogonal bond cleavage of a 3D **rht** MOF. (b) Schematic of how the 3D **rht BCN-231** can be orthogonally cleaved on its alkene bonds to synthesize cuboctahedral MOPs with different external functionalities. (c) Cleavage, by ozonolysis, of the three olefinic bonds of L_1_ needed to release the MOPs from the 3D framework. When the ozonolysis is performed using reductive workup (DMS = dimethyl sulfide, as reducing agent), all the olefinic bonds of the framework are selectively cleaved into aldehyde groups.

To demonstrate the feasibility of this synthesis, we initially selected the 3D MOF **PCN-61** as our reticular precursor.^43^ Within this **rht**-Cu-MOF, cuboctahedral Cu_24_bdc_24_ cavities (where bdc= 1,3-benzenedicarboxylate)^44^ spontaneously form during the assembly of Cu(II) ions and the hexacarboxylate linker 5,5′,5″-benzene-1,3,5-triyltris(1-ethylnyl-2-isophthalate) (btei); a linker composed of three bdc moieties connected by a central phenyl ring at its 1, 3, and 5 positions via alkyne bonds. Each bdc moiety of btei contributes to the formation of a distinct cuboctahedral cavity, resulting in the 3D interconnection of different cavities in **PCN-61** through the central alkyne-benzene unit of this linker, which acts as a trigonal (3-c) symmetric node. Based on this structure, we reasoned that cleavage of the three alkyne bonds of btei through ozonolysis would release the cavities in the form of cuboctahedral Cu(II)-based MOPs (Figure 1). However, despite subjecting **PCN-61** to various ozonolysis conditions, we were unable to quantitatively cleave those alkyne bonds, which precluded us from synthesizing the isolated MOPs (Figure S1). Moreover, ozonolysis of alkynes requires the presence of water in the media,^45^ which, as we observed, also promotes partial hydrolysis of the labile Cu-COO bonds.^46^

To enhance bond cleavage within this 3D structure using ozone, we chose to substitute all alkyne bonds in **PCN-61** with alkene bonds. This modification ensured that the cuboctahedral Cu_24_bdc_24_ cavities in the new structure would now be linked through alkene bonds, a type of bond that requires less aggressive conditions than alkynes to be ozonized and that we recently demonstrated could be quantitatively cleaved in reticular materials using ozone (Figure 1).^21−24^ Additionally, controlling the workup steps after ozonolysis at low temperature can lead to the homolytic cleavage of alkene bonds.^47^ Consequently, this control could facilitate the Clip-off synthesis of Cu_24_bdc_24_ cages or MOPs featuring only one type of functional group on the outer surface: for example, we envisioned that using reductive workup conditions would afford 24 aldehyde groups (Figure S2).

To this end, we initially synthesized the new hexacarboxylate linker 1,3,5-tris[5-(*E*)-vinylisophthalic acid]benzene (referred to as H_6_L_1_; Figure 1 and Figures S3–S5). Subsequently, crystals of the isoreticular **rht**-Cu-MOF (hereafter denoted as **BCN-231**) were obtained by reacting the new linker with Cu(NO_3_)_2_·2.5H_2_O in *N,N*-dimethylformamide (DMF) at 70 °C for 48 h (Figure 2a, left). Single-crystal X-ray diffraction (SCXRD) confirmed the formation of the expected Cu-MOF exhibiting an underlying **rht-**topology. Within it, the assembled cuboctahedral cavities, reminiscent of cuboctahedral Cu_24_bdc_24_ MOPs, were periodically spaced through alkene-benzene units (Figure 1). Powder X-ray diffraction (PXRD) and N_2_ sorption measurements validated the phase purity of **BCN-231** and revealed a remarkably high Brunauer–Emmett–Teller surface area (*S*_BET_) of 3139 m^2^ g^–1^ (Figures S6–S11).

**Figure 2:**
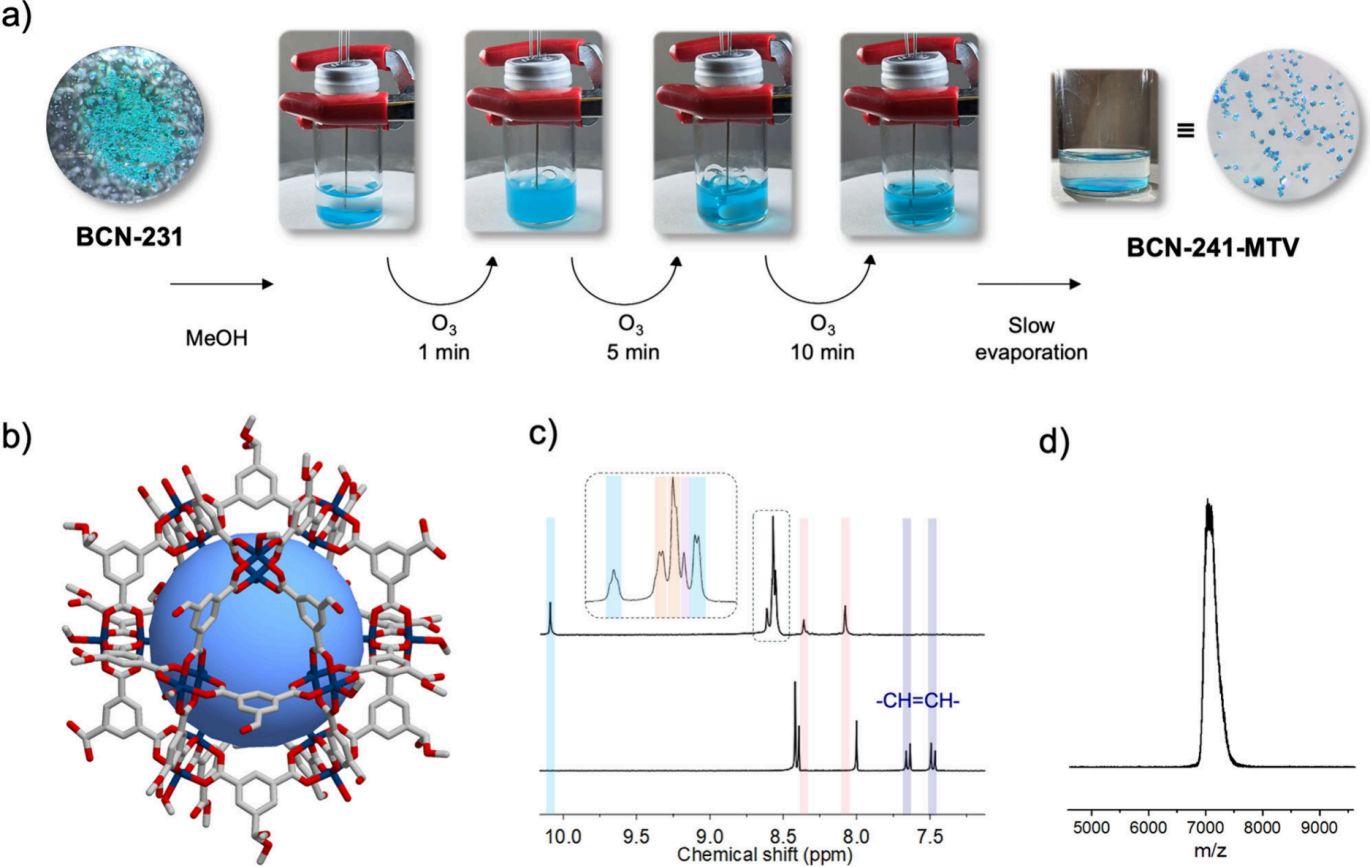
(a) Photographs of the synthesis of **BCN-241-MTV** via Clip-off Chemistry. From left to right: starting with a crystalline sample of **BCN-231**; introduction of these crystals into methanol; bubbling ozone into the methanolic dispersion, causing the cleavage of L_1_; “dissolution” of **BCN-231** and release of the cuboctahedral MOPs; and finally, crystallization of cuboctahedral MOPs (Video S1). (b) SCXRD structure of **BCN-241-MTV**. Hydrogen atoms have been omitted for clarity. Cu(II): blue, O: red, C: gray. (c) ^1^H NMR spectra (DMSO-*d*_6_/DCl) of digested **BCN-241-MTV** (top) and **BCN-231** (bottom). Note the lack of alkene bond (violet) from initial **BCN-231**, and the displacement of bdc signals owing to the formation of **BCN-241-MTV** functionalized with aldehyde (coming from 5-formylisophthalic acid, blue), acetal (coming from 5-(dimethoxymethyl)isophthalic acid, red), carboxylic acid (coming from benzene-1,3,5-tricarboxylic acid, purple), and ester groups (coming from 5-(methoxycarbonyl)isophthalic acid, orange). (d) MALDI-ToF-MS spectrum of **BCN-241-MTV**.

Having prepared **BCN-231**, we next proceeded with cleavage of its olefinic bonds. To this end, it was dispersed in methanol, and the resultant solution was treated with ozone at a constant flux (30 g Nm^–3^) at room temperature. The disconnection and formation of MOP species became evident to the naked eye, as the solid suspension transitioned into a transparent blue solution within 10 min (Figure 2a). The ozonolysis reaction was monitored using ultraviolet–visible spectroscopy (UV–vis) and matrix-assisted laser desorption/ionization-time-of-flight mass spectrometry (MALDI-ToF-MS), with periodic analysis of the supernatant at t = 1, 5, and 10 min (Figures S12 and S13). UV–vis analysis of the solution revealed the presence of a broad band centered at 700 nm, characteristic of Cu(II) paddlewheel clusters, indicating that these clusters were not disassembled during the reaction.^48^ Moreover, the MALDI-ToF-MS spectrum contained a broad peak ranging from approximately 5440 to 6856 *m*/*z*, consistent with the formation of a cuboctahedral Cu(II)-based MOP within the first minute of the reaction. Then, after ozonolysis, a blue solid was rapidly precipitated from the supernatant by the addition of ether and characterized by proton nuclear magnetic resonance (^1^H NMR). ^1^H NMR spectrum revealed the complete disappearance of alkene signals from **BCN-231** (δ = 7.66, 7.63, 7.49, and 7.46 ppm, Figure S14). It also revealed the emergence of new signals, which we attributed to a mixture of different ozonolysis products, including 5-formylisophthalic acid, 5-(dimethoxymethyl)isophthalic acid, benzene-1,3,5-tricarboxylic acid, and 5-(methoxycarbonyl)isophthalic acid, resulting from the uncontrolled oxidative cleavage of L_1_ in methanol (Figures S2 and S14). Altogether, these results confirmed the selective cleavage of the alkene bonds, the release of the MOP from the framework, and the stability of the “released” MOP in solution.

Next, we attempted to crystallize the product by slowly evaporating off the solvent from the above ozonolysis product under ambient conditions, which afforded blue crystals (yield = 79%) suitable for SCXRD (Figure 2a, right). The SCXRD data confirmed formation of a multivariate cuboctahedral Cu_24_bdc_24_ MOP (hereafter denoted as **BCN-241-MTV**), comprising 12 Cu–Cu paddlewheel clusters connected through 24 5-substituted bdc linkers (Figure 2b). The external surface of each cage was decorated with a mixture of the aforementioned functional groups (aldehydes, acetals, carboxylic acids and esters) at the 5-position of the linker. According to SCXRD data, potentially disordered groups at the 5-position were approximated as follows: 10 aldehydes, 4 acetals, 2 carboxylic acids, and 8 esters. To further investigate the composition of the external surface of these cages, we analyzed the crystals of **BCN-241-MTV** by NMR. To this end, the crystals were digested in DMSO-*d*_6_/DCl, and the products from three independent reactions were studied by ^1^H NMR (Figure 2c, Figure S14). The average values for the external functional group distribution for **BCN-241-MTV** were: 9.6 ± 1.8 aldehydes, 4.1 ± 1.7 acetals, 1.4 ± 1.2 carboxylic acids, and 9.0 ± 2.8 esters (Table S3), a ratio similar to that observed in the single-crystal structure. MALDI-ToF-MS analysis of **BCN-241-MTV** revealed a broad peak ranging from 6860.3 to 7374.3 *m*/*z* (Figure 2d, Figure S15), which includes the mass of the single MOP cage with the average functional group composition and six DMF solvent molecules (expected: 7052.6 *m*/*z*). Interestingly, nitrogen-sorption measurements on **BCN-241-MTV** demonstrated its microporosity, revealing a N_2_ uptake of 133 cm^3^ g^–1^ at P/P_0_ = 0.95 and a *S*_BET_ of up to 425 m^2^ g^–1^. (Figure 3d, Figures S16–S20).

**Figure 3:**
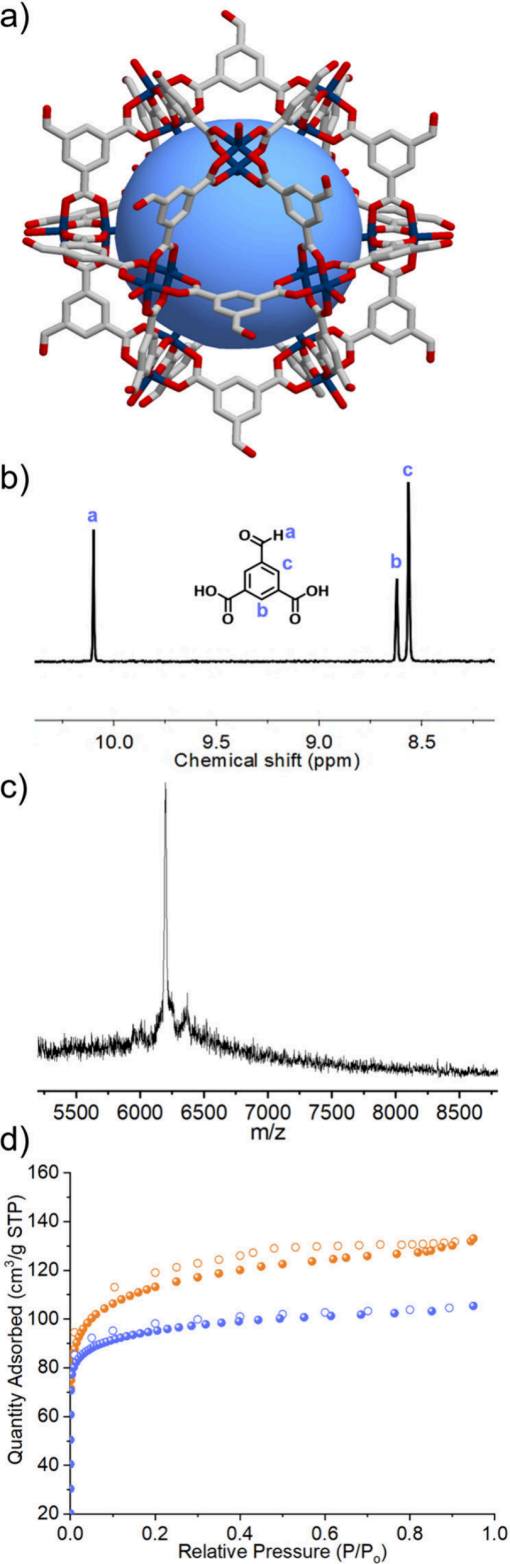
(a) SCXRD structure of **BCN-241-CHO**, showing the 24 aldehyde groups on the external surface of the MOP. Hydrogen atoms have been omitted for clarity. Cu(II): blue, O: red, C: gray. (b) ^1^H NMR spectrum (DMSO-*d*_6_/DCl) of digested **BCN-241-CHO**, showing exclusively the peaks corresponding to 5-formylisophthalic acid. (c) MALDI-ToF-MS spectrum of **BCN-241-CHO**. (d) N_2_ isotherms (77 K) for **BCN-241-MTV** (orange) and **BCN-241-CHO** (blue).

Having demonstrated our ability to orthogonally cleave bonds in **BCN-231** and subsequently isolate the unconnected cuboctahedral MOPs, we next endeavored to control the synthesis of the cuboctahedral MOPs whose 24 external functional groups were exclusively aldehydes (hereafter denoted as **BCN-241-CHO**), using reductive conditions. For this, we dispersed **BCN-231** in methanol and exposed the resulting dispersion to a constant ozone flux (30 g Nm^–3^) for 10 min at −78 °C. This yielded a blue solution, into which was added dimethyl sulfide (DMS) as reducing agent. UV–vis analysis of the solution confirmed no degradation of the paddlewheel clusters upon DMS addition (Figure S21). Afterward, diethyl ether was added, and after 12 h, the resultant solution afforded blue crystals (yield = 62%).

SCXRD analysis of these crystals revealed the successful synthesis of the cuboctahedron-shaped Cu(II)-based cage functionalized with 24 aldehyde groups (Figure 3a). The exclusive presence of aldehyde groups on the surface of the MOP synthesized via Clip-off Chemistry was further confirmed by ^1^H NMR of the acid-digested (DMSO-*d*_6_/DCl) MOP product. In addition to the expected disappearance of olefinic protons of **BCN-231** at δ = 7.66, 7.63, 7.49, and 7.46 ppm, characteristic signals for 5-formylisophthalic acid (δ = 10.16, 8.68, and 8.62 ppm) were clearly identifiable (Figure 3b and Figure S22). Formation of **BCN-241-CHO** was also corroborated by MALDI-ToF-MS, where a sharp peak at 6200.7 *m*/*z* was observed, consistent with the expected molecular weight of [Cu_24_(CHO-bdc)_24_+H^+^]^+^ · 2 MeOH (*m*/*z*= 6200.6) (Figure 3c, Figure S23). Furthermore, the phase purity of the sample was confirmed by the close match between the experimental and simulated PXRD patterns for **BCN-241-CHO** (Figure S24). Finally, N_2_-sorption measurements also showed this MOP to be porous, with a N_2_ uptake of 105.4 cm^3^ g^–1^ at P/P_o_ = 0.95 and a *S*_BET_ of 368 m^2^ g^–1^ (Figure 3d, Figures S25–S29).

In summary, we have reported the first-ever controlled synthesis and isolation of a metal–organic finite structure, such as a cage or MOP, through selective cleavage of olefinic bonds within a 3D MOF. By employing orthogonal olefinic bond cleavage, and optimizing the required ozonolysis, we successfully synthesized, via Clip-off Chemistry, two distinctly functionalized cuboctahedral cages. These findings underscore the versatility and potential applications of Clip-off Chemistry in synthesizing novel finite complex supramolecular architectures (*e*.*g*., cages, catenanes, etc.) that can be derived from the structures found within the vast array of reported 2 and 3D MOFs.

## Supporting Information Available

Detailed synthesis and methods, NMR spectra, UV–vis spectra, mass spectrometry analysis, crystallographic data, FT-IR spectra, FE-SEM images, TGA analysis, PXRD diffractograms, and nitrogen adsorption–desorption isotherms (PDF)
Video showing the ozonolysis reaction for the clip-off synthesis of the cuboctahedral Cu(II)-based MOP (MP4)
