# Supplementary material for: Synthesis of Metal–Organic Cages via Orthogonal Bond Cleavage in 3D Metal–Organic Frameworks
Source: J Am Chem Soc. 2024 Sep 23;146(39):26603–8. doi: 10.1021/jacs.4c09431 (PMC11450890; doi:10.1021/jacs.4c09431)
Supplement: Supplementary file 1 — ja4c09431_si_001.pdf [file ja4c09431_si_001.pdf]

Electronic Supporting information for:

# Synthesis of Metal-Organic Cages via Orthogonal Bond Cleavage in 3D Metal-Organic Frameworks

Sara Ruiz-Relaño,[a,b] Dongsik Nam,[a,b] Jorge Albalad,*[a,b] Alba Cortés-Martínez,[a,b] Judith Juanhuix,[c] Inhar Imaz,*[a,b] and Daniel Maspoch*[a,b,d]

[a] Catalan Institute of Nanoscience and Nanotechnology (ICN2), CSIC and Barcelona Institute of Science and Technology, Campus UAB, Bellaterra, 08193 Barcelona, Spain.

[b] Departament de Química, Facultat de Ciències, Universitat Autònoma de Barcelona, Campus UAB, Bellaterra, 08193 Barcelona, Spain.

[c] ALBA Synchrotron Light Facility, Cerdanyola del Vallès, 08290 Barcelona, Spain.

[d] ICREA, Passeig Lluis Companys 23, 08010 Barcelona, Spain.

# Table of Contents

**Section S1. General Methods and Materials** ........ S3

**S1.1. Chemicals and reagents** ........ S3

**S1.2. Instruments** ........ S3

**Section S2. Synthetic Procedures** ........ S5

**S2.1. Synthesis of 1,3,5-tris[5-(*E*)-vinylisophthalic acid]benzene ($H_6L_1$)** ........ S5

**S2.2. Synthesis of BCN-231** ........ S6

**S2.3. Synthesis of BCN-241-MTV via Clip-off Chemistry** ........ S6

**S2.4. Synthesis of BCN-241-CHO via Clip-off Chemistry** ........ S6

**Section S3. Supplementary Figures** ........ S7

**Section S4. $H_6L_1$** ........ S9

**Section S5. BCN-231** ........ S12

**Section S6. BCN-241-MTV** ........ S19

**Section S7. BCN-241-CHO** ........ S30

**References** ........ S40

## Section S1. General Methods and Materials

### S1.1. Chemicals and reagents

All the reagents and solvents were purchased from commercial sources and used without further purification unless otherwise specified. Copper (II) nitrate hemi(pentahydrate) ($Cu(NO_3)_2 \cdot 2.5\ H_2O$), dimethyl sulfide (DMS), potassium hydroxide (KOH), triethyl phosphite ($P(OEt)_3$), 1,3,5-triformylbenzene, and potassium *tert*-butoxide ($^t$BuOK) were purchased from Sigma-Aldrich (Merck). Toluene (99.5% HPLC grade) was purchased from Labkem. Anhydrous tetrahydrofuran (THF), ethanol (EtOH), methanol (MeOH), hydrochloric acid 36% *v/v* (HCl), *N'N*-dimethylformamide (DMF), ethyl acetate (EtOAc), and diethyl ether ($Et_2O$) were purchased from Fisher Scientific. Dimethyl 5-(bromomethyl)isophthalate was purchased from BLD Pharm. Deuterium chloride (DCl) 20 wt. % in $D_2O$ was purchased from Acros Organics. Deuterated dimethyl sulfoxide (DMSO-$d_6$) was purchased from Eurisotop. Deionized water was obtained with a Milli-Q® filter system (18.2 MΩ·cm).

### S1.2. Instruments

**Single-Crystal X-Ray Diffraction (SCXRD).** Crystallographic data for **BCN-231**, **BCN-241-MTV** and **BCN-241-CHO** was collected at 100 K at the XALOC beamline at ALBA synchrotron light facility (λ= 0.82653 Å).[1] Data were indexed, integrated, and scaled using the Xia2 and programs that Xia2 used.[2-7] Absorption correction was not applied. The structures were solved by direct methods and subsequently refined by correction of F2 against all reflections, using SHELXT2018 within Olex2 package.[8,9] All non-hydrogen atoms were refined with anisotropic thermal parameters by full-matrix least-squares calculations on F2 using the program SHELXL2018.[10] The hydrogen atoms were calculated in their expected positions with the HFIX instruction of SHELXL2018 and refined as riding atoms with Uiso(H) = 1.5 Ueq(C). We treated the presence of solvent molecules in the cavities of all structures running solvent mask using Olex2 solvent mask.[9-11] In **BCN-231**, we counted 7391 electrons per unit cell that correspond to 185 DMF molecules. In **BCN-241-MTV**, we counted 1993 electrons per unit cell that correspond to 111 methanol molecules. Finally, the 2697 electrons masked in **BCN-241-CHO** are consistent with 64 molecules of diethyl ether used to crystalize the crystals. The thermal motions of the aldehyde, ester, and acetal groups within **BCN-241-MTV** were restrained with ISOR and SIMU.

**Powder X-Ray Diffraction (PXRD)** data were recorded on an X'Pert PRO MPD analytical diffractometer (Panalytical) at 45 KV, 40 mA using Cu $K_\alpha$ radiation (λ = 1.5418 Å). Synchrotron PXRD patterns were collected at the BL13-XALOC beamline at the ALBA synchrotron. Experiments were carried out using a monochromatic X-ray beam with a wavelength of λ = 0.82653 Å.[1] Data were collected using a Pilatus3 X 6M detector. The powder diffraction patterns were radially integrated (circle coordinates) using FIT2D program.[12]

**Proton ($^1H$) Nuclear Magnetic Resonance (NMR)** spectra were collected on a Bruker Avance NEO 300 MHz and a Bruker Avance NEO 400 MHz spectrometers at the Servei de Ressonància Magnètica Nuclear from the Autonomous University of Barcelona (SeRMN-UAB). Digestion of **BCN-231**, **BCN-241-MTV** and **BCN-241-CHO** samples were carried out by dissolving approximately 5 mg of sample in a mixture of 500 µL of DMSO-$d_6$ and 10 µL DCl (20 wt. % in $D_2O$).

**Matrix Assisted Laser Desorption/Ionization-Time of Flight (MALDI-ToF) mass spectrometry (MS)** spectra were acquired on an Applied Biosystems 4700 Proteomics Analyzer at the Centres Científics i Tecnològics from the University of Barcelona (CCiTUB). Samples were measured using *trans*-2-[3-(4-tert-butylphenyl)-2-methyl-2-propenylidene]malononitrile (DCTB) as a matrix and positive ionization mode.

**Electrospray ionization Time of Flight (ESI-ToF) mass spectrometry (MS)** measurements were performed using a LC/MSD-TOF (Agilent Technologies, G1969A) in negative mode, frag. 175 V at the Centres Científics i Tecnològics from the University of Barcelona (CCiTUB).

**Ozonolysis** was carried out using an ozone generator GHBZO3-E Commercial Ozone Generator from ZonoSistem equipped with ozone analyzer UVOZ-1200.

**Fourier Transform Infrared (FT-IR)** spectra were acquired of a Bruker Tensor 27FT-IR spectrometer equipped with a Golden Gate diamond attenuated total reflection (ATR) cell. All spectra were collected neatly in ambient atmosphere.

**Field Emission Scanning Electron Microscopy (FE-SEM)** imaging was collected on a SEM Quanta 650FEG. The samples were fully dried before measuring.

**Thermogravimetric analysis (TGA)** curves were carried on a Pyris Perkin Elmer TGA 8000 under nitrogen atmosphere and heating rate of 5 ºC/min.

**Ultraviolet-visible (UV-Vis)** spectra were acquired using a Thermo Scientific™ NanoDrop 200 at room temperature (*ca*. 25 ºC).

**Volumetric $N_2$** measurements were collected at 77 K using an ASAP 2460 (Micromeritics) and High-Resolution ASAP 2020 (Micromeritics). Temperatures for isotherms measurement were controlled by using a liquid nitrogen bath. The specific surface area ($S_{BET}$) values were calculated according to the BETSI method.[13]

**Supercritical $CO_2$ drying** was performed using Laboratory Supercritical Fluid Equipment SFE15 mL (Extratex Supercritical Fluid Innovation).

## Section S2. Synthetic Procedures

### S2.1. Synthesis of 1,3,5-tris[5-(*E*)-vinylisophthalic acid]benzene ($H_6L_1$)

*Synthesis of dimethyl 5-((dietoxyphosphoryl)methyl)isophthalate* (**1**).

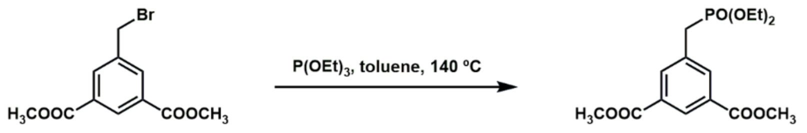


1

Dimethyl 5-(bromomethyl)isophthalate (500 mg, 1.7 mmol) and toluene (10 mL) were added to a 25 mL Schlenk flask equipped with a magnetic stirrer and a rubber stopper. The mixture was then bubbled with argon for 15 minutes, and triethyl phosphite (1.5 mL, 6.5 mmol) was added. Then, the mixture was stirred overnight at 140 ºC. The resulting solution was dried under reduced pressure until a colorless oil was obtained. To remove the excess of triethyl phosphite, a five-cycle co-evaporation process was performed by adding EtOH (5 x 20 mL) and boiling at 120 ºC. Cooling the oil to room temperature afforded the formation of **1** as a white solid (460 mg, yield= 91%). $^1$H NMR (300 MHz, DMSO-$d_6$): $\delta$ (ppm)= 8.37 (s, 1H), 8.16 (s, 2H), 3.97 (m, 4H), 3.91 (s, 6H), 3.51 (s, 1H), 3.48 (s, 1H), 1.17 (t, 6 H).

*Synthesis of 1,3,5-tris[5-(E)-vinylisophthalic acid]benzene* (**$H_6L_1$**).

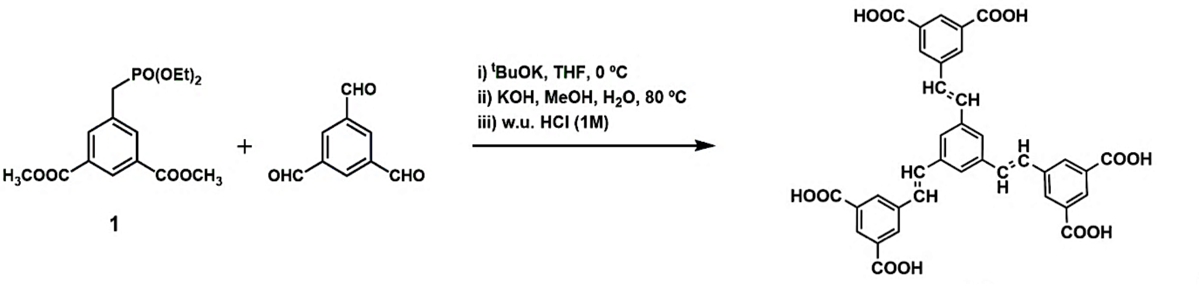


Potassium *tert*-butoxide (203.8 mg, 1.8 mmol) was dispersed in anhydrous THF (6 mL) under argon atmosphere, and slowly added to a solution of 1,3,5-triformylbenzene (49.1 mg, 0.3 mmol) and **1** (600 mg, 1.8 mmol) in anhydrous THF (6 mL) at 0 ºC, previously degassed with argon. The pale orange mixture was stirred overnight at room temperature. The reaction evolution was followed by thin layer chromatography (TLC) (EtOAc:MeOH 9:1) until 1,3,5-triformylbenzene was consumed. $H_2O$ (20 mL) was then added, and the mixture was concentrated under vacuum to remove the THF. The obtained solid was recovered by filtration and mixed with MeOH (20 mL) and an aqueous solution of KOH (238.4 mg, 4.3 mmol in 5 mL of distilled water), and refluxed for 24 hours at 80 ºC. The solution was dried under reduced pressure until a white solid was obtained. The solid was redissolved in the minimum amount of $H_2O$ and precipitated by the dropwise addition of HCl (1 M) up to pH= 1. The resulting pale-yellow solid was filtered, washed with distilled water (10 mL x 3), MeOH/$Et_2O$ (10 mL x 3) and dried

under vacuum (132 mg, yield= 67%). $^{1}$H NMR (300 MHz, DMSO-$d_6$): $\delta$ (ppm)= 13.40 (s, 6H), 8.42 (s, 6H), 8.40 (s, 3H), 8.00 (s, 3H), 7.66-7.63 (d, 3H), 7.49-7.46 (d, 3H).

**S2.2. Synthesis of BCN-231**

$Cu(NO_3)_2 \cdot 2.5\ H_2O$ (268.6 mg, 1.2 mmol) and **$H_6L_1$** (65.0 mg, 0.1 mmol) were dissolved in DMF (5 mL) in a 23 mL scintillation vial. Then, 6M hydrochloric acid (0.3 mL) was added to the solution. The vial was heated in a preheated oven at 70 ºC for 48 hours. After cooling to room temperature, the resulting teal-colored crystals were washed with DMF (3 x 10 mL) and exchanged with MeOH (3 x 10 mL) (120.8 mg, yield= 88% based on **$H_6L_1$**).

**S2.3. Synthesis of BCN-241-MTV via Clip-off Chemistry**

25 mg of **BCN-231** were dispersed in 2 mL of MeOH, and placed into a glass vial equipped with a magnetic stirrer sealed with a plastic cap. A constant ozone flux ($O_3$ = 30.0 g $Nm^{-3}$) was bubbled into the dispersion through a syringe for 10 minutes at room temperature, resulting in a blue solution. Afterwards, the solution was bubbled with argon for 2 minutes to remove excess ozone. Slow evaporation of the solution at room temperature afforded single-crystals of **BCN-241-MTV**. The crystals were washed with MeOH three times (yield= 79 %).

**S2.4. Synthesis of BCN-241-CHO via Clip-off Chemistry**

25 mg of **BCN-231** were dispersed in 2 mL of MeOH, and placed into a glass vial equipped with a magnetic stirrer sealed with a plastic cap. A constant ozone flux ($O_3$ = 30.0 g $Nm^{-3}$) was bubbled into the dispersion through a syringe for 10 minutes at -78 ºC (dry ice/acetone bath), resulting in a blue solution. The solution was bubbled with argon for 2 minutes to remove excess ozone. Then, 100 µL of DMS were added, and the mixture was stirred for 75 minutes at room temperature. Afterwards, $Et_2O$ was poured into the blue solution (≈ 20 mL), affording single-crystals of **BCN-241-CHO** after 12 hours. The crystals were washed with $Et_2O$ three times (yield= 62 %).

## Section S3. Supplementary Figures

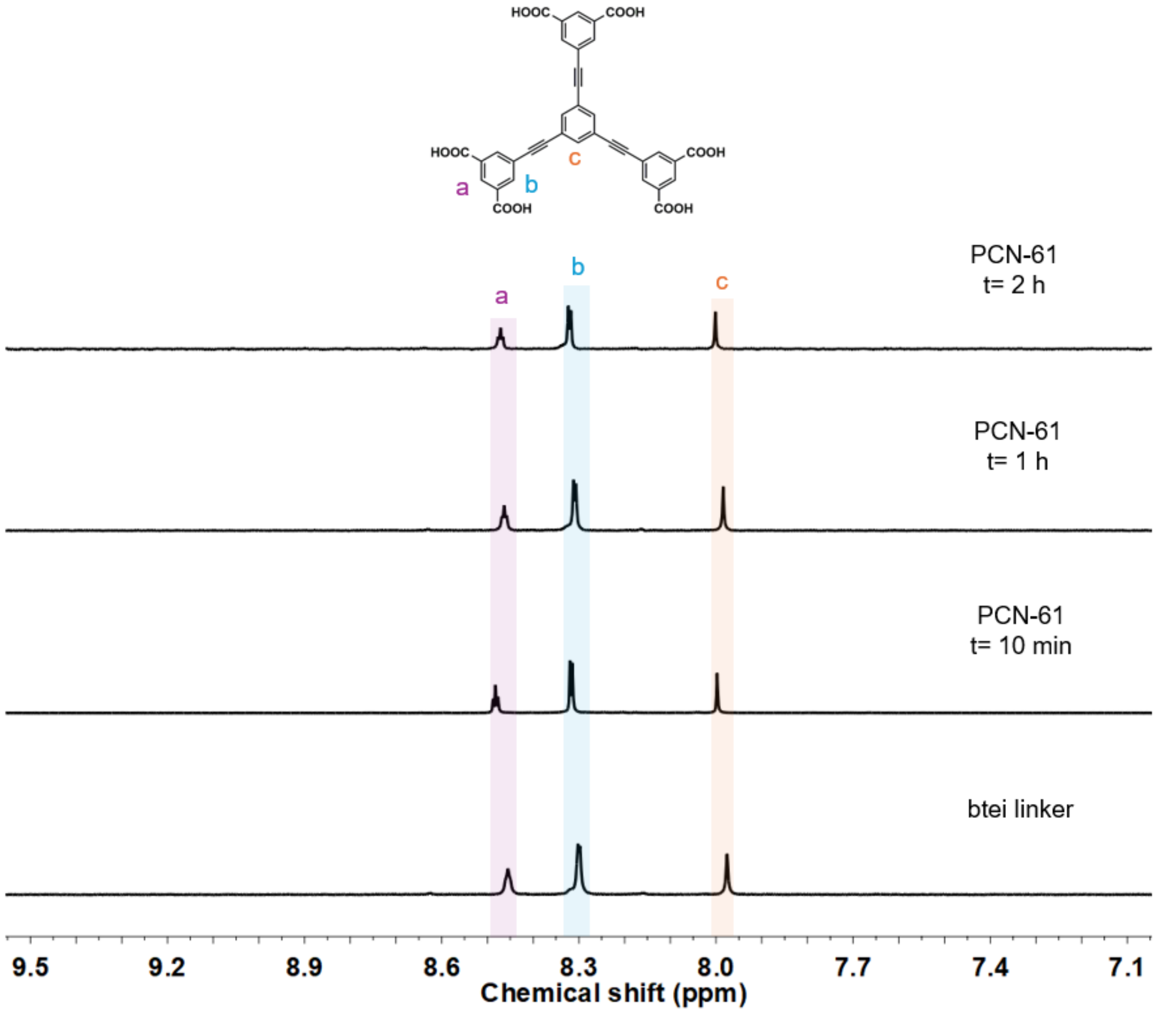


**Figure S1.** $^{1}$H NMR (300 MHz, DMSO-$d_6$) spectrum of btei linker (bottom). $^{1}$H NMR (300 MHz, DMSO-$d_6$/DCl) spectra of **PCN-61** after being exposed to ozone at three time intervals (t= 10 min,

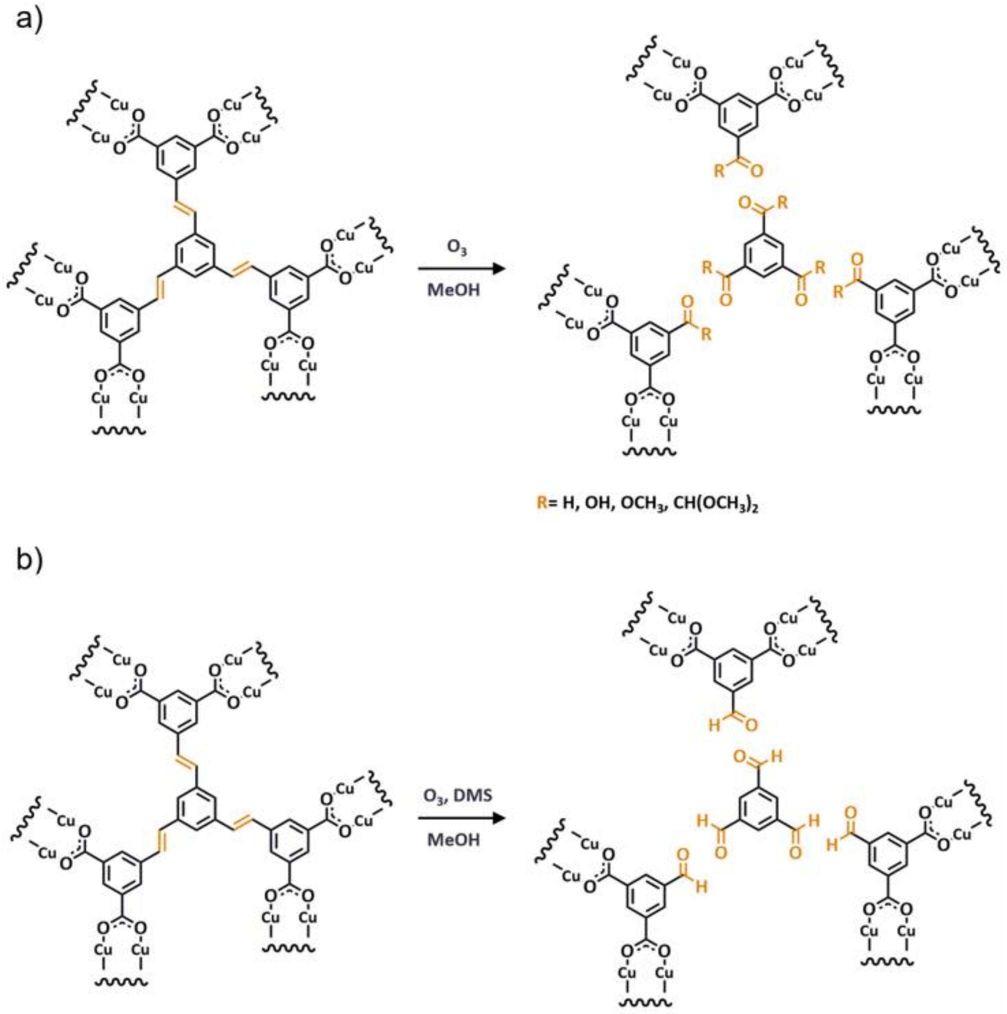


**Figure S2.** Schematic representation of ozonolysis reaction, selectively cleaving alkene bonds in methanol. a) Ozonolysis reaction without further work-up step produces a mixture of aldehyde, carboxylic acid, ester, and acetal functional groups. b) Ozonolysis reaction under reductive work-up leads the reaction towards obtaining aldehyde groups as the exclusive cleavage product.

## Section S4. $H_6L_1$

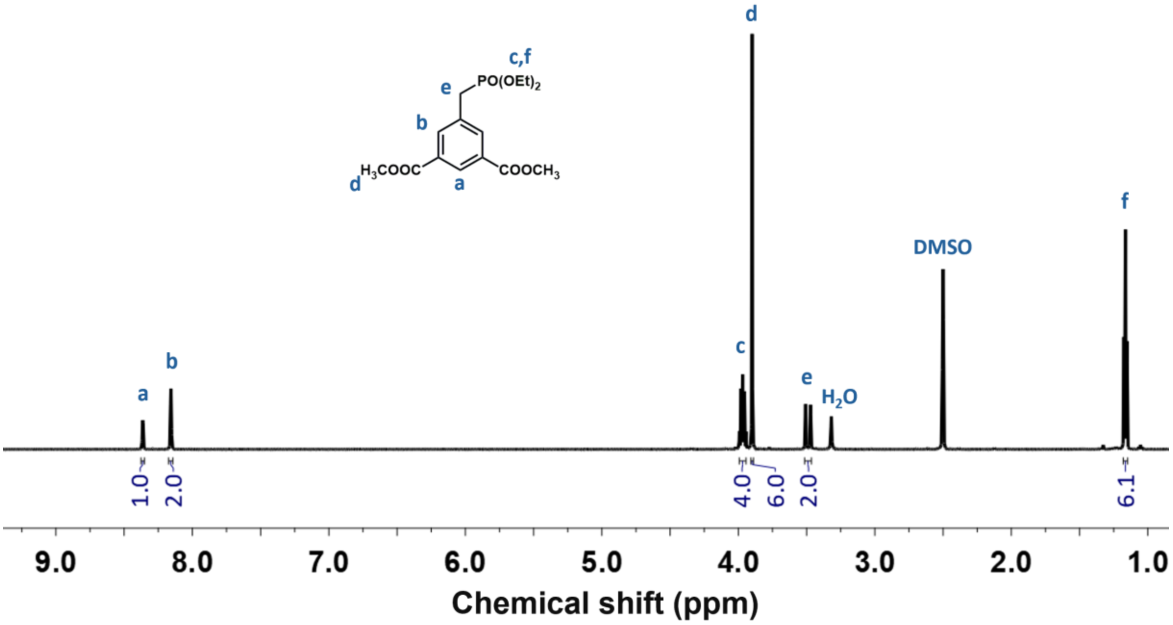


**Figure S3.** $^{1}$H NMR (300 MHz, DMSO-$d_6$) spectrum of intermediate **1**.

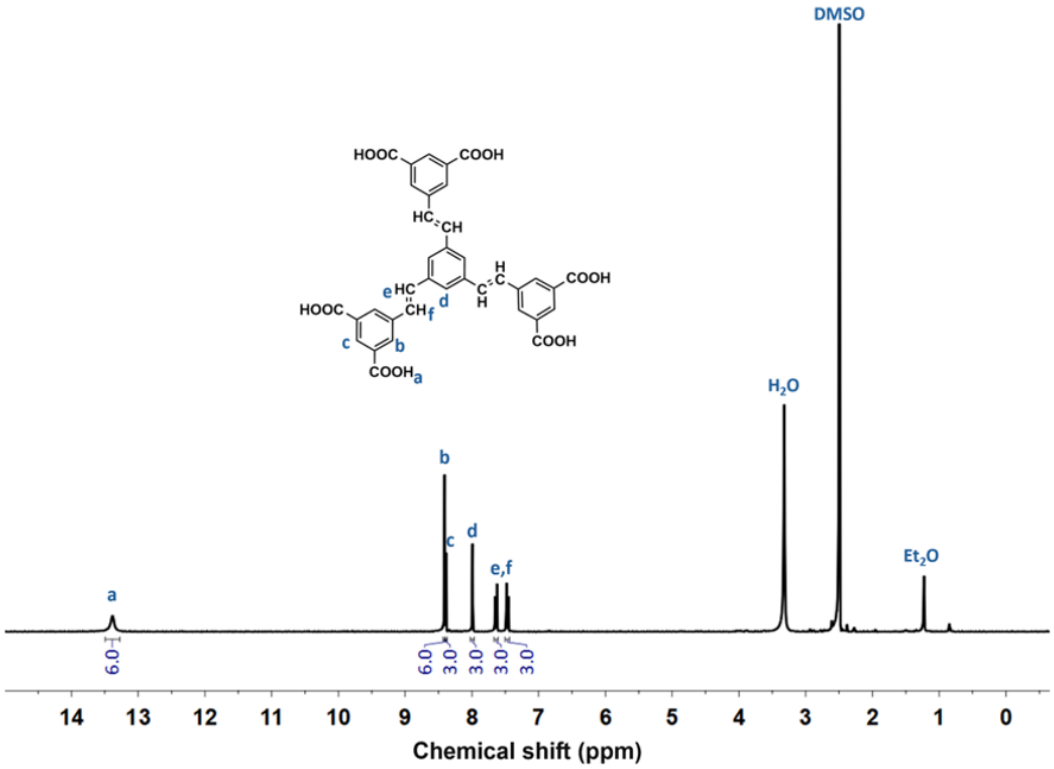


**Figure S4.** $^1$H NMR (300 MHz, DMSO-d$_6$) spectrum of of **$H_6L_1$** linker.

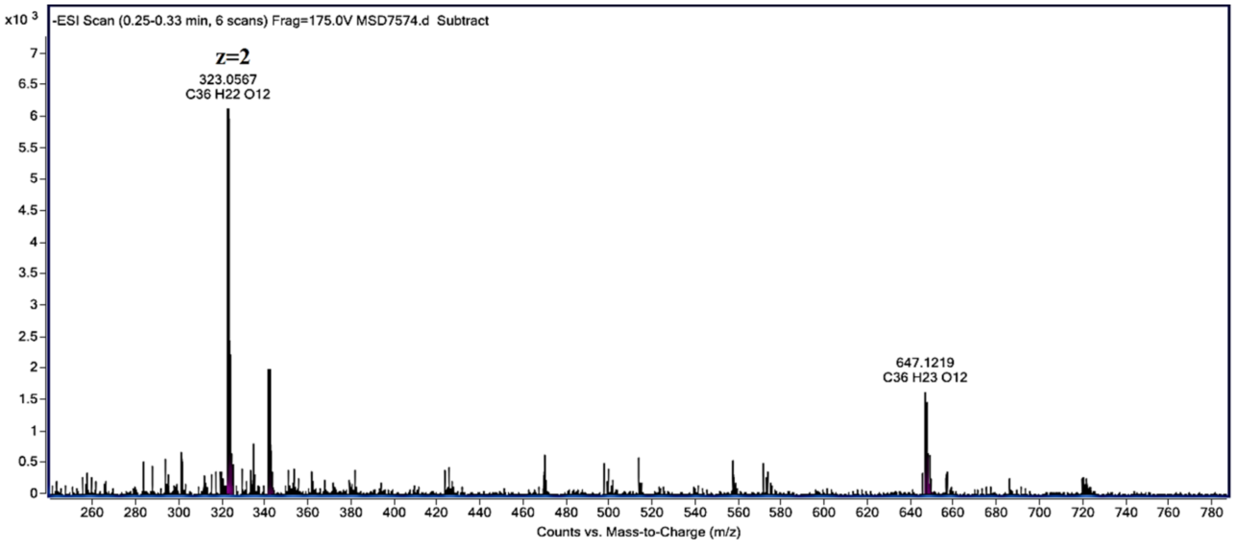


**Figure S5**. ESI-MS spectrum of $\mathbf{H_6L_1}$ linker. Peaks observed at 647.1 and 323.1 m/z correspond to $[H_6L_1 - H^+]^-$ and $[H_6L_1 - 2H^+]^{2-}$, with theoretical m/z of 647.1 and 323.1, respectively.

# Section S5. BCN-231

**Table S1.** Crystal data and structure refinement for **BCN-231**.

| | |
|---|---|
| CCDC number | 2367002 |
| Empirical formula | $C_{73}H_{36}Cu_6O_{29}$ |
| Formula weight | 1758.26 |
| Temperature/K | 100 |
| Crystal system | Tetragonal |
| Space group | *I*4/*m* |
| a/Å | 29.66495(8) |
| b/Å | 29.66495(8) |
| c/Å | 42.0838(2) |
| α/° | 90 |
| β/° | 90 |
| γ/° | 90 |
| Volume/Å$^3$ | 37034.1(3) |
| Z | 8 |
| $\rho_{calc}$g/cm$^3$ | 0.631 |
| μ/mm$^{-1}$ | 1.069 |
| F(000) | 7040.0 |
| Crystal size/mm$^3$ | 0.07 × 0.07 × 0.06 |
| Radiation/Å | Synchrotron (λ = 0.82653) |
| 2Θ range for data collection/° | 2.258 to 62.206 |
| Index ranges | 0 ≤ h ≤ 37, 0 ≤ k ≤ 37, 0 ≤ l ≤ 51 |
| Reflections collected | 193579 |
| Independent reflections | 17928 [$R_{int}$ = 0.1043, $R_{sigma}$ = 0.0535] |
| Data/restraints/parameters | 17928/0/493 |
| Goodness-of-fit on F$^2$ | 1.249 |
| Final R indexes [I>=2σ (I)] | $R_1$ = 0.0554, w$R_2$ = 0.1789 |
| Final R indexes [all data] | $R_1$ = 0.0626, w$R_2$ = 0.1814 |
| Largest diff. peak/hole / e Å$^{-3}$ | 1.15/-0.65 |

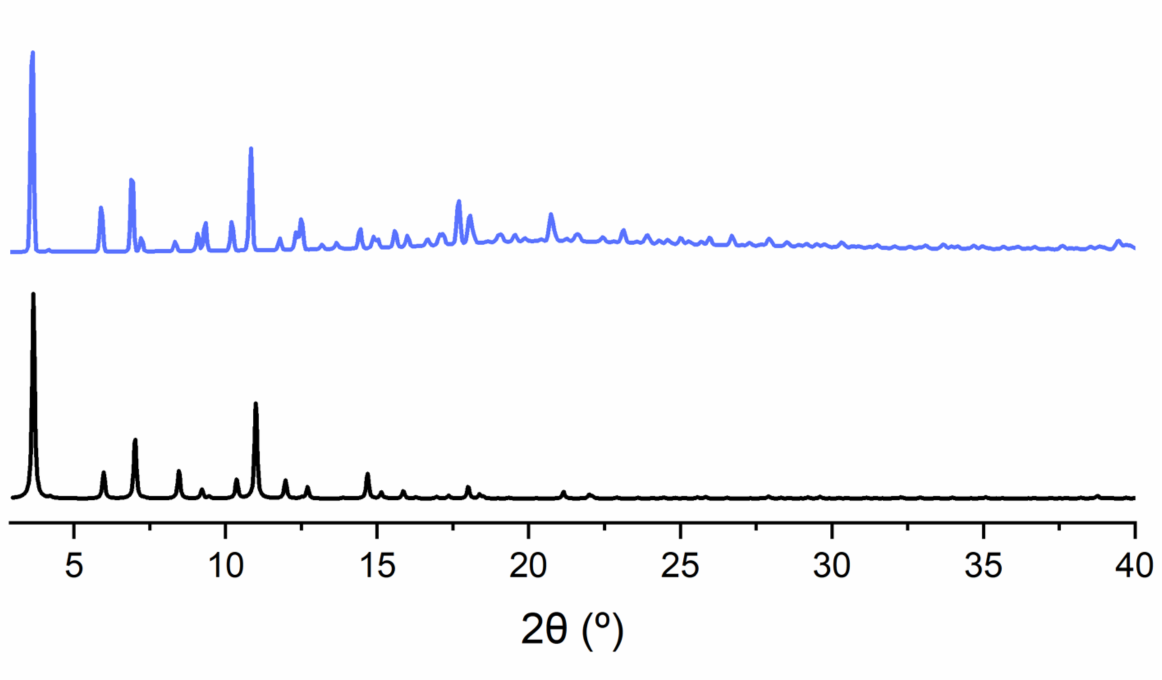


**Figure S6.** PXRD patterns of as-synthesized (blue) and simulated **BCN-231** (black).

For $N_2$ gas adsorption, 30 mg of **BCN-231** were washed with DMF and then, MeOH for 2 days. Afterwards, **BCN-231** was activated by supercritical $CO_2$ drying for 2 hours. Volumetric $N_2$ (77 K) isotherm was recorded after further activating the sample at 65 ºC under vacuum.

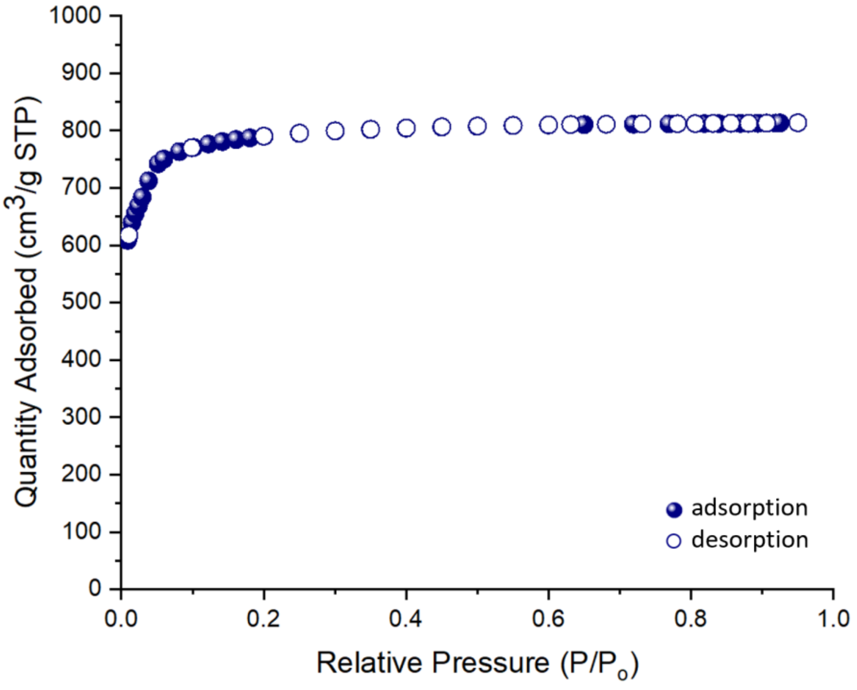


**Figure S7.** $N_2$ adsorption isotherm of **BCN-231** at 77 K.

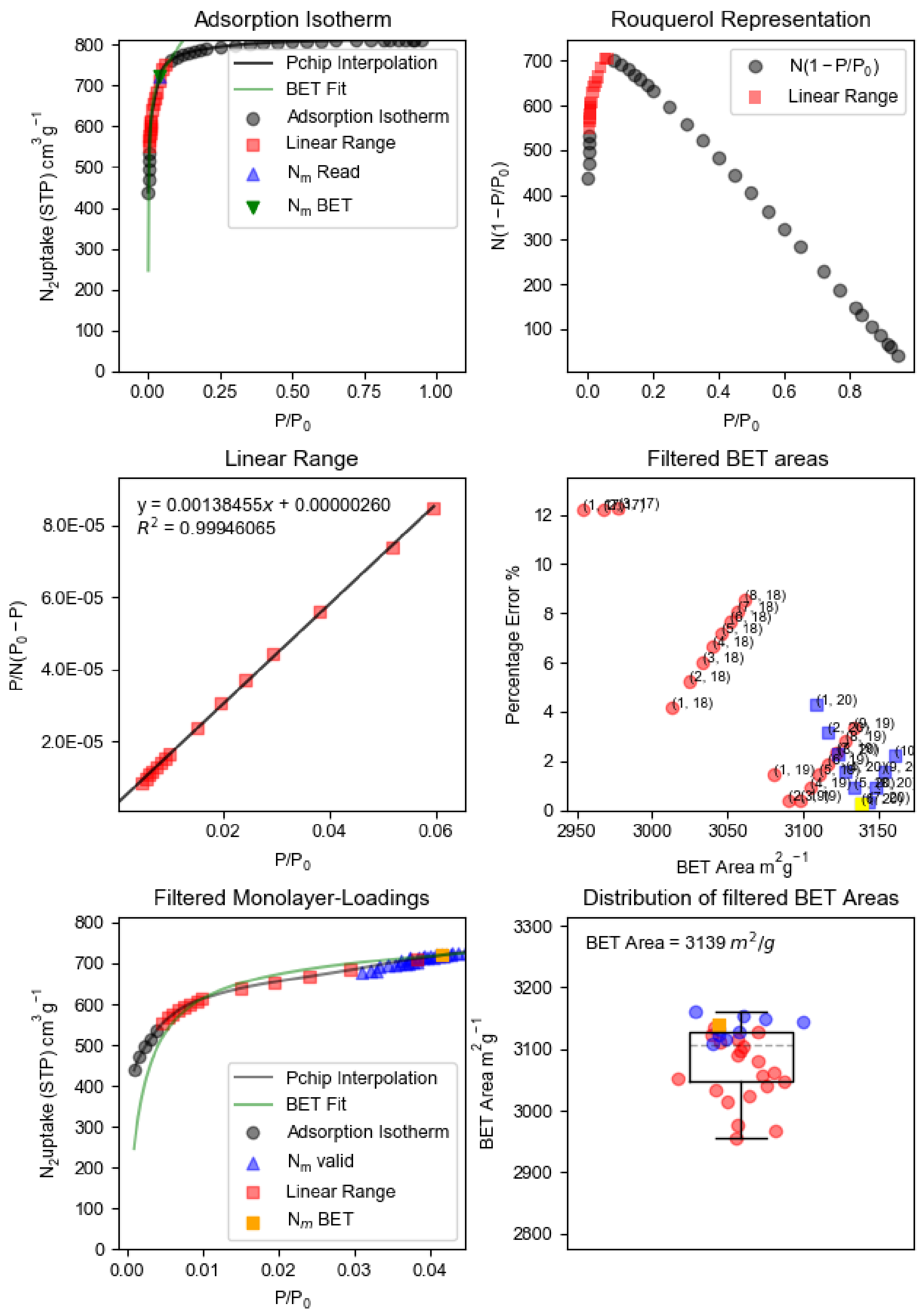


**Figure S8.** BETSI analysis of **BCN-231** ($S_{BET}$ = 3139 $m^2g^{-1}$).

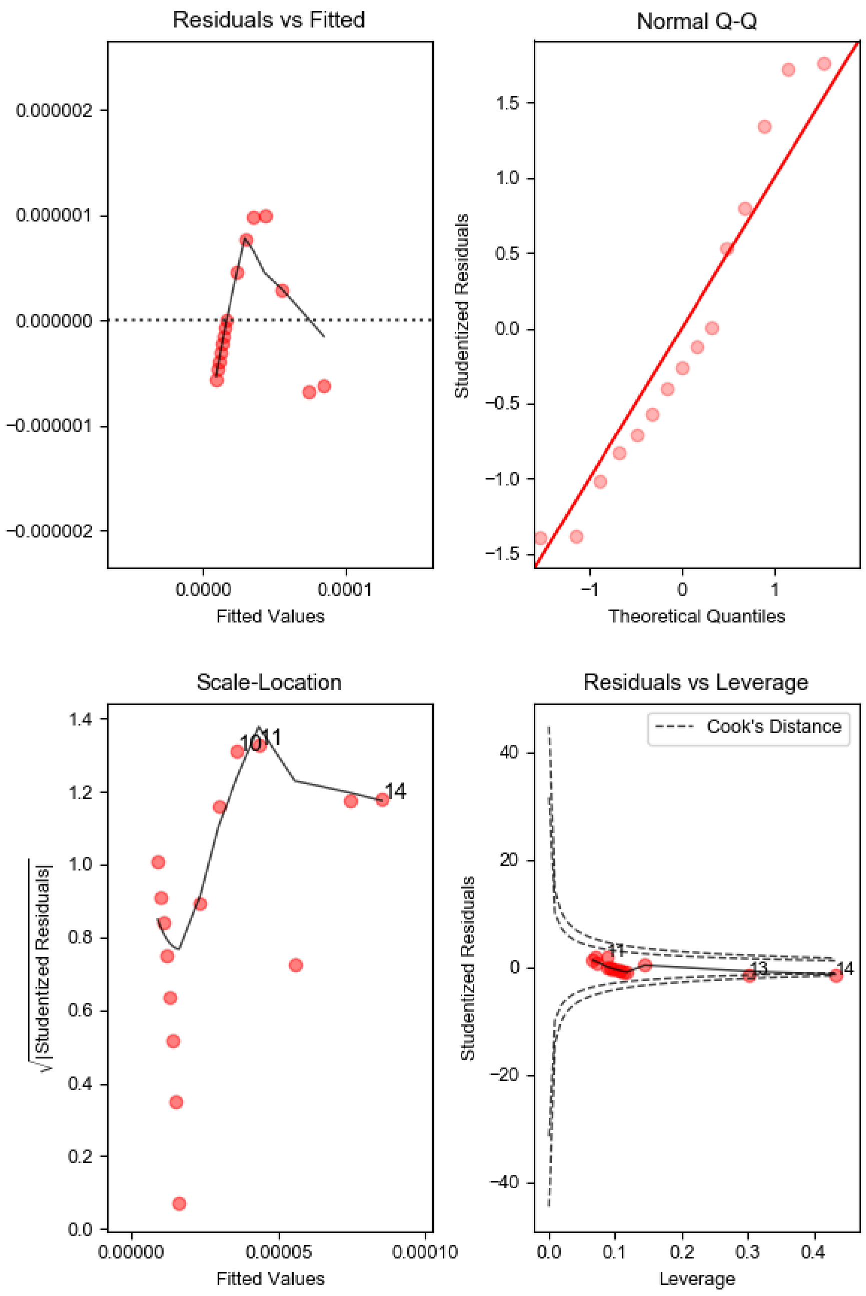


**Figure S9.** BETSI regression diagnostics of **BCN-231**.

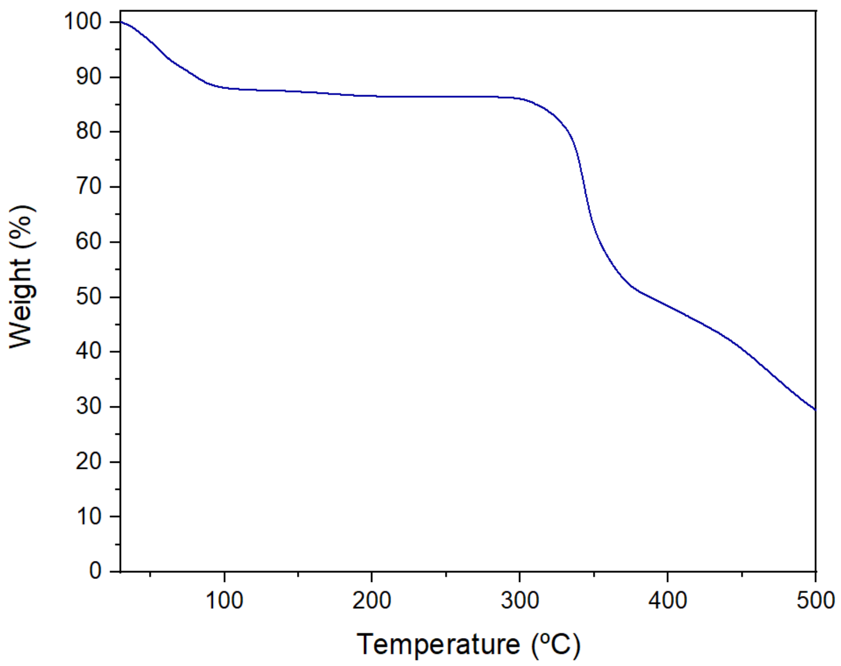


**Figure S10.** Thermogravimetric analysis of **BCN-231**. Note that the weight loss step around 60 ºC is attributed to free and coordinated solvent molecules. The decomposition temperature of **BCN-231** above 300 ºC agrees to reported values for Cu(II)-paddlewheel-based structures.[14-15]

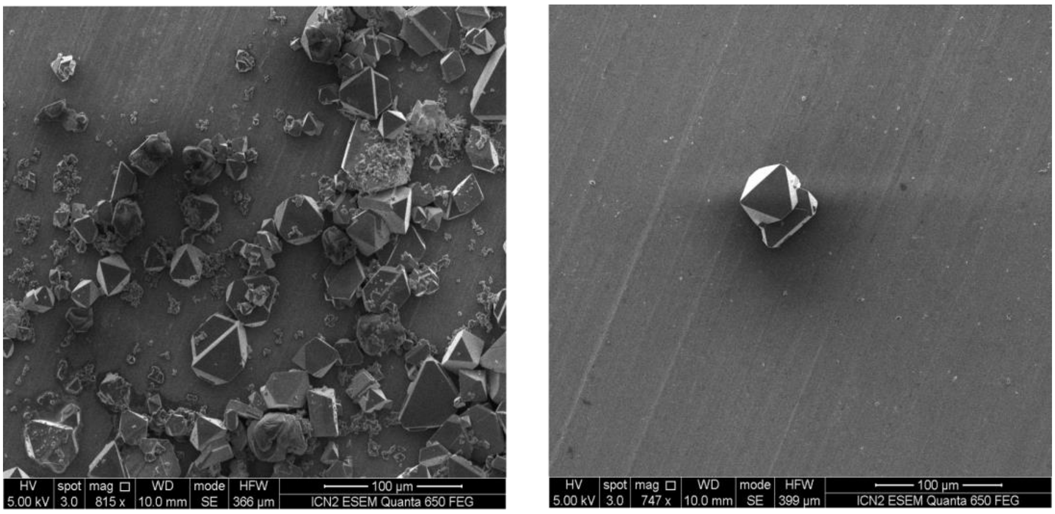


**Figure S11.** FE-SEM images of crystals of **BCN-231**.

## Section S6. BCN-241-MTV

**Table S2.** Crystal data and structure refinement for **BCN-241-MTV**.

| | |
|---|---|
| CCDC number | 2366265 |
| Empirical formula | $C_{242}H_{254}Cu_{24}O_{183}$ |
| Formula weight | 7615.40 |
| Temperature/K | 100 |
| Crystal system | Monoclinic |
| Space group | *C*2/*m* |
| a/Å | 24.767 |
| b/Å | 43.907 |
| c/Å | 24.330 |
| α/° | 90 |
| β/° | 119.35 |
| γ/° | 90 |
| Volume/Å$^3$ | 23061.7 |
| Z | 2 |
| $\rho_{calc}$g/cm$^3$ | 1.097 |
| μ/mm$^{-1}$ | 1.738 |
| F(000) | 7732 |
| Crystal size/mm$^3$ | 0.10 × 0.09 × 0.06 |
| Radiation/Å | Synchrotron (λ = 0.82653) |
| 2Θ range for data collection/° | 2.234 to 49.942 |
| Index ranges | -25 ≤ h ≤ 20, 0 ≤ k ≤ 44, 0 ≤ l ≤ 24 |
| Reflections collected | 80313 |
| Independent reflections | 12471 [$R_{int}$ = 0.1066, $R_{sigma}$ = 0.0933] |
| Data/restraints/parameters | 12471/237/1050 |
| Goodness-of-fit on F$^2$ | 2.49 |
| Final R indexes [I>=2σ (I)] | $R_1$ = 0.1759, w$R_2$ = 0.4415 |
| Final R indexes [all data] | $R_1$ = 0.1981, w$R_2$ = 0.4582 |

**BCN-241-MTV** could have disorders of aldehyde, carboxylic acid, and ester groups due to their overlapping geometries. However, because of the limited data quality, we approximated the composition by assuming a single type of functional group at each 5-position in the single-crystal structure. Consequently, the composition (10 aldehydes, 4 acetals, 2 carboxylic acids, and 8 esters) was proposed to understand the approximate structure of **BCN-241-MTV**. Each cage within the same crystal will have a different distribution of functional groups.

UV-Vis analysis was used to confirm the presence of Cu(II)-paddlewheel during ozonolysis reaction. The aliquots (100 µL) from ozonolysis were diluted with 300 µL of methanol and measured *in-situ* at three different times (t= 1, 5, and 10 minutes) at room temperature.

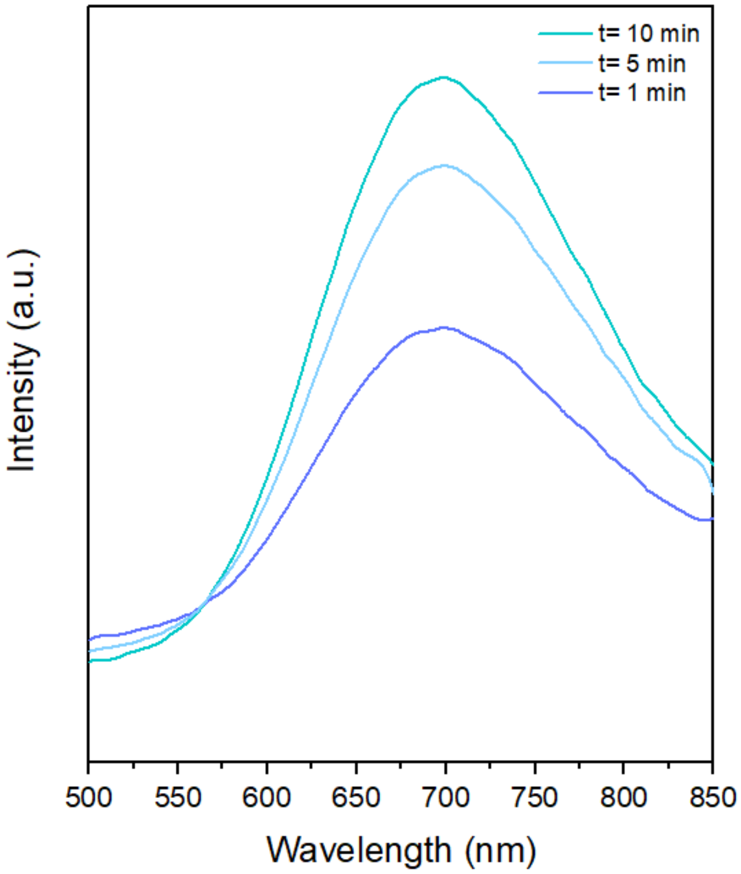


**Figure S12.** UV-Vis spectra of the supernatant at three different reaction times (t= 1, 5 and 10 minutes). Note the presence of a broad band centered at 700 nm, characteristic of Cu(II) paddlewheel clusters.[16] Note also that the intensity of the band was increased over the reaction time due to the release and dissolution of more **BCN-241-MTV** to the reaction solution.

MALDI-ToF analysis of **BCN-241-MTV** was carried out by taking three aliquots (≈ 100 µL) at t = 1, 5, 10 minutes during the ozonolysis reaction of **BCN-231**. The aliquots were diluted with 400 µL of methanol, mixed in a 1:1 ratio with a stock solution of a DCTB matrix in dichloromethane (10 mg/mL), drop-casted on the measuring plate, and dried prior to analysis. Samples were measured in positive ionization mode.

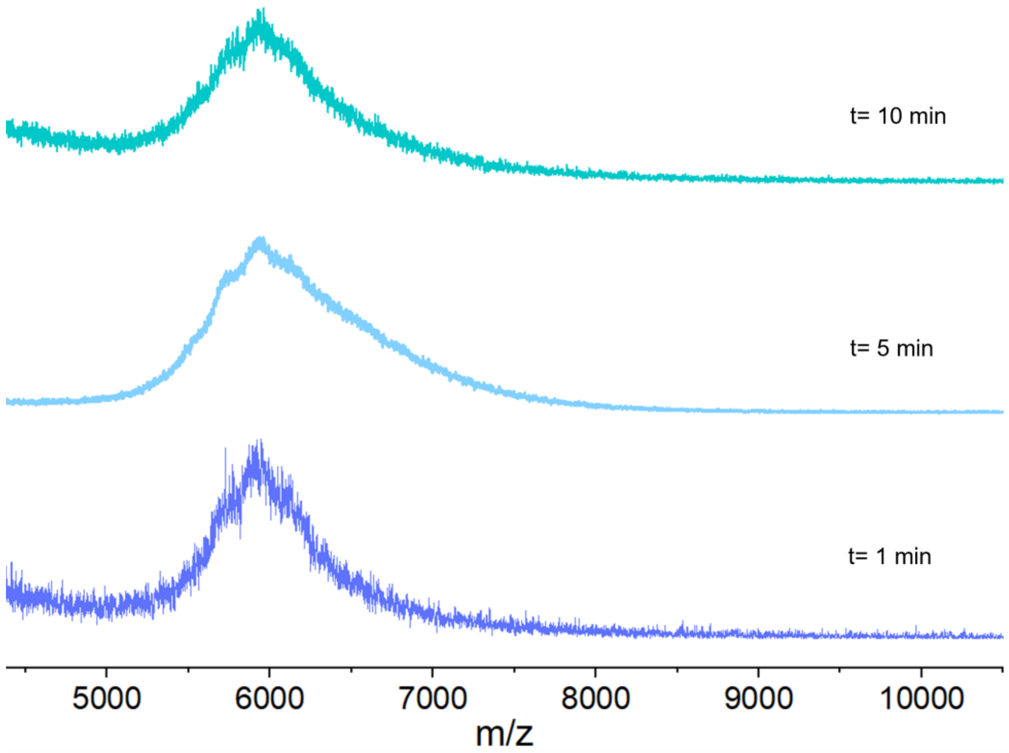


**Figure S13.** MALDI-ToF analysis of the supernatant during the ozonolysis reaction in MeOH. Note the presence of a broad peak (5440 - 6856 m/z) for the three different reaction times (t= 1, 5 and 10 minutes). The presence of this peak is consistent with the formation of a cuboctahedral Cu(II)-based MOP functionalized with aldehyde, ester, carboxylic acid and acetal groups, as later confirmed by SCRXD.

$^{1}$H NMR measurement of **BCN-241-MTV** was carried out by digesting 10 mg of the sample with 10 μL of DCl (20 wt. % solution in $D_2O$) in 600 μL of DMSO-$d_6$.

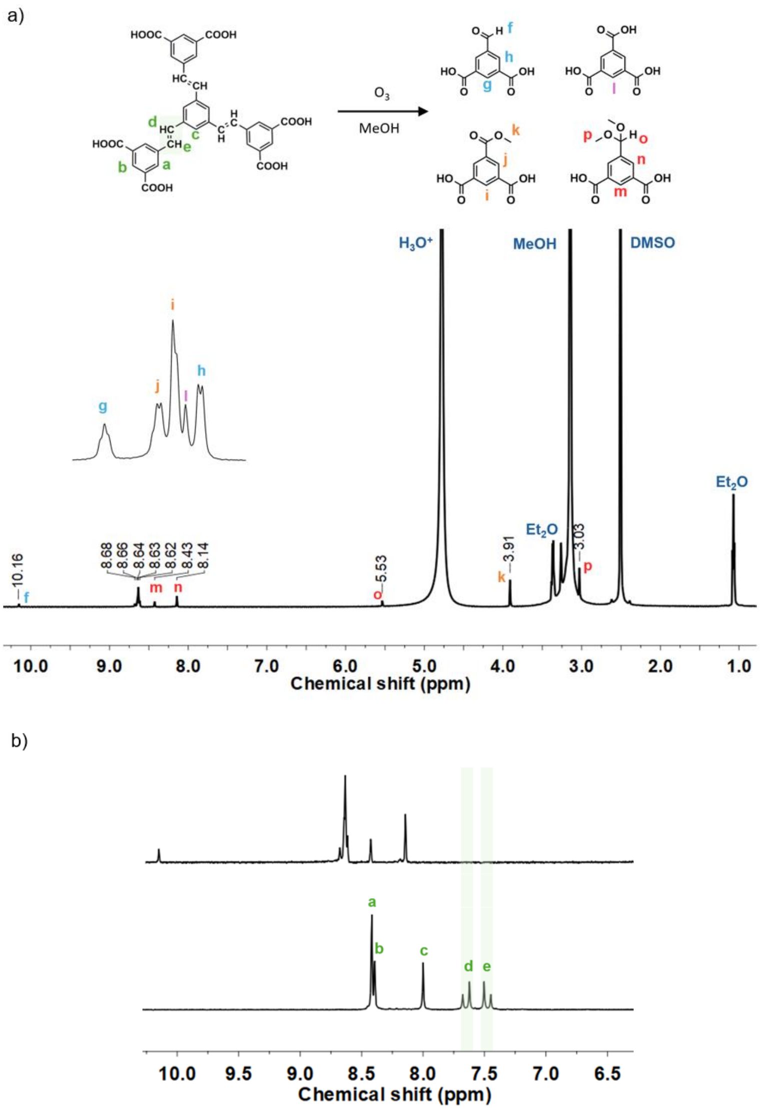


**Figure S14.** a) $^{1}$H NMR (300 MHz, DMSO-$d_6$/DCl) spectrum of the digested blue solid precipitated with $Et_2O$ from the reaction product, showing the new signals attributed to a mixture of different ozonolysis products: 5-formylisophthalic acid (δ= 10.16, 8.68, and 8.62 ppm, blue), 5-(methoxycarbonyl)isophthalic acid (δ= 8.66 and 8.64 ppm, orange), benzene-1,3,5-tricarboxylic acid (δ= 8.63 ppm, purple), and 5-(dimethoxymethyl)isophthalic acid (δ= 8.43, 8.14, 5.53, and 3.03 ppm, red). Residual solvent peaks were not eliminated due to the potential susceptibility of acetal groups to decompose under elevated temperatures/vacuum. b) $^{1}$H NMR (300 MHz, DMSO-$d_6$/DCl) spectra of digested **BCN-241-MTV** crystals (top) and **BCN-231** (bottom). Note the lack of olefinic peaks (δ= 7.66, 7.63, 7.49, 7.46 ppm, green) after ozonolysis.

**Table S3.** Ratio of functional groups (5-formylisophthalic acid, 5-(methoxycarbonyl)isophthalic acid, benzene-1,3,5-tricarboxylic acid, and 5-(dimethoxymethyl)isophthalic acid) found by $^{1}$H-NMR in **BCN-241-MTV** (from three different synthesized samples).

| | 5-formyl isophthalic acid | 5-(methoxycarbonyl) isophthalic acid | benzene-1,3,5-tricarboxylic acid | 5-(dimethoxymethyl) isophthalic acid |
|---|---|---|---|---|
| Sample 1 | 10.96 | 6.30 | 0.5 | 5.8 |
| Sample 2 | 7.6 | 11.92 | 0.83 | 3.89 |
| Sample 3 | 10.3 | 8.67 | 2.8 | 2.5 |
| Standard deviation | 1.78 | 2.82 | 1.24 | 1.67 |
| Average | 9.6 ± 1.8 | 9.0 ± 2.8 | 1.4 ± 1.2 | 4.1 ± 1.7 |

For MALDI-ToF measurement, the obtained crystals of **BCN-241-MTV** were washed three times with MeOH and dissolved in DMF. Afterwards, the DMF solution was diluted in 400 µL of methanol, mixed in a 1:1 ratio with a stock solution of a DCTB matrix in dichloromethane (10 mg/mL), drop-casted on the measuring plate, and dried prior to analysis. Samples were measured in positive ionization mode.

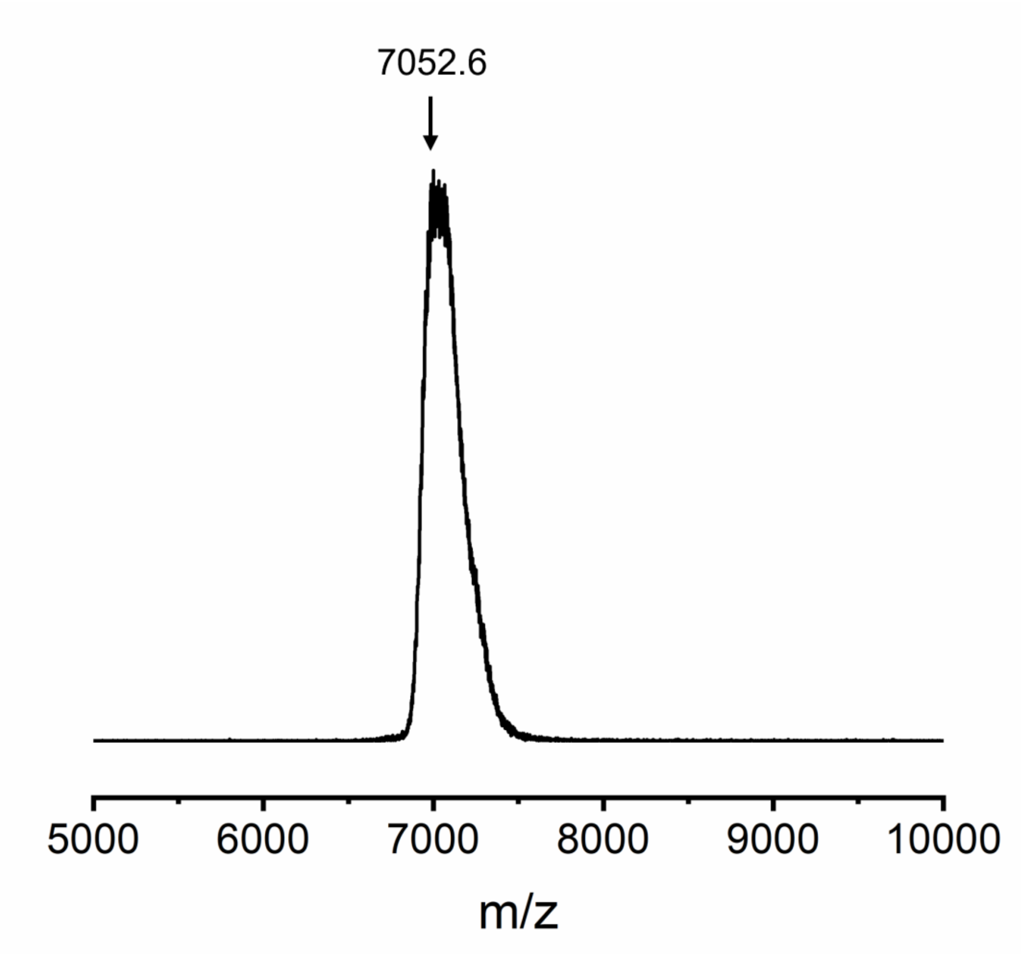


**Figure S15.** MALDI-ToF spectrum of **BCN-241-MTV** in DMF. The broad peak ranging from 6860.3 - 7374.3 includes the mass [BCN-241-MTV + $H^+$]$^+$· 6 DMF (m/z= 7052.6).

For $N_2$ gas adsorption of **BCN-241-MTV**, 20 mg of sample was washed with MeOH three times. Afterwards, **BCN-241-MTV** was activated by supercritical $CO_2$ drying for 2 hours, and volumetric $N_2$ (77 K) isotherm was recorded after activating the sample at room temperature in vacuum.

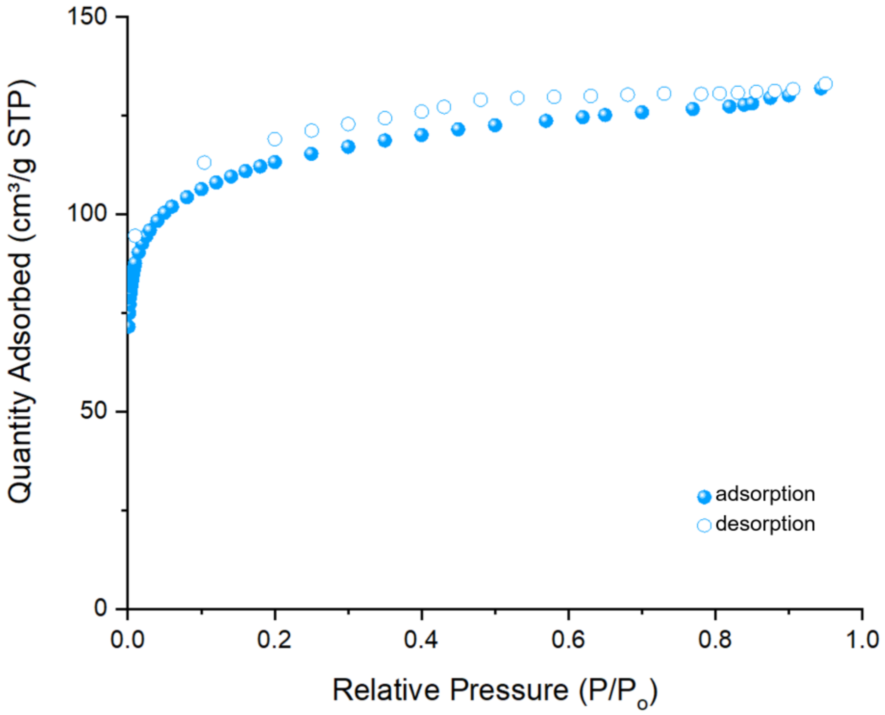


**Figure S16.** $N_2$ adsorption isotherm of **BCN-241-MTV**.

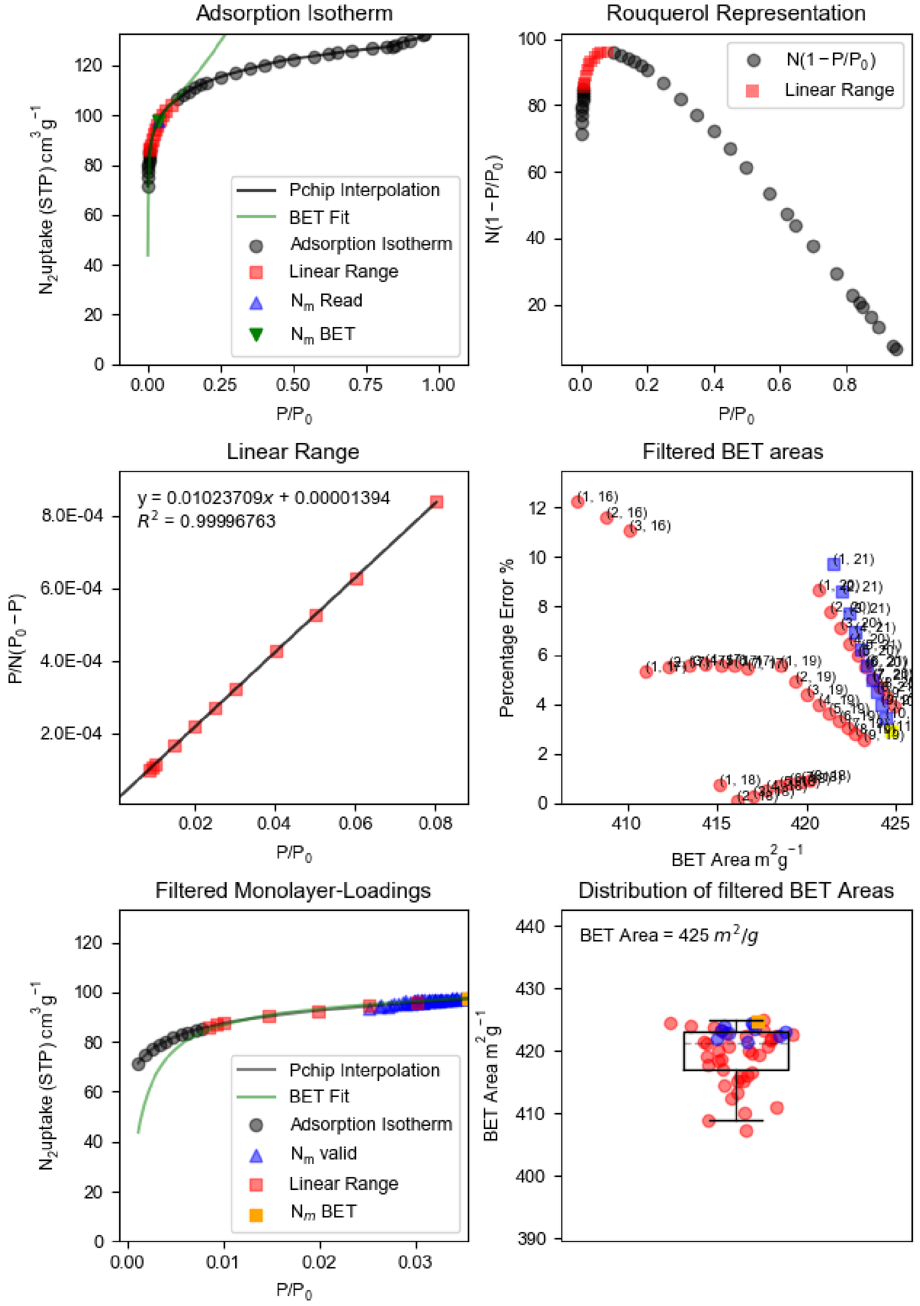


**Figure S17.** BETSI analysis of **BCN-241-MTV** ($S_{BET}$ = 425 $m^2g^{-1}$).

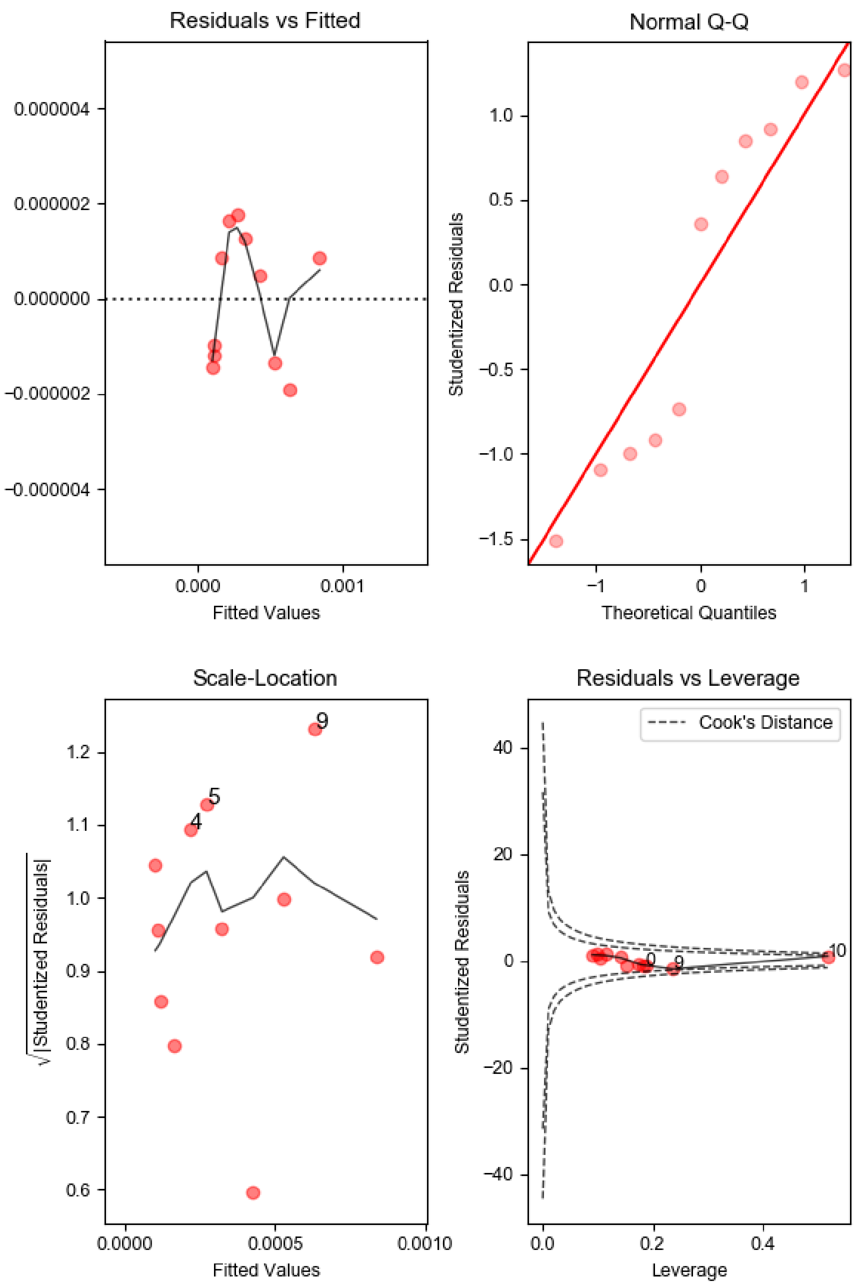


**Figure S18.** BETSI regression diagnostics of **BCN-241-MTV**.

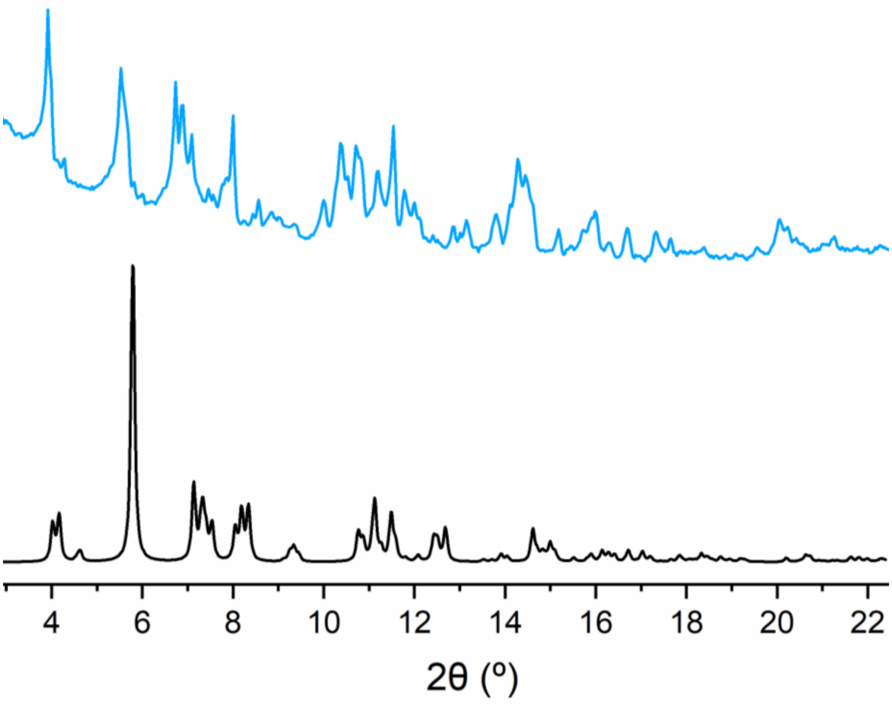


**Figure S19.** PXRD patterns of as-synthesized (blue) and simulated **BCN-241-MTV** (black).

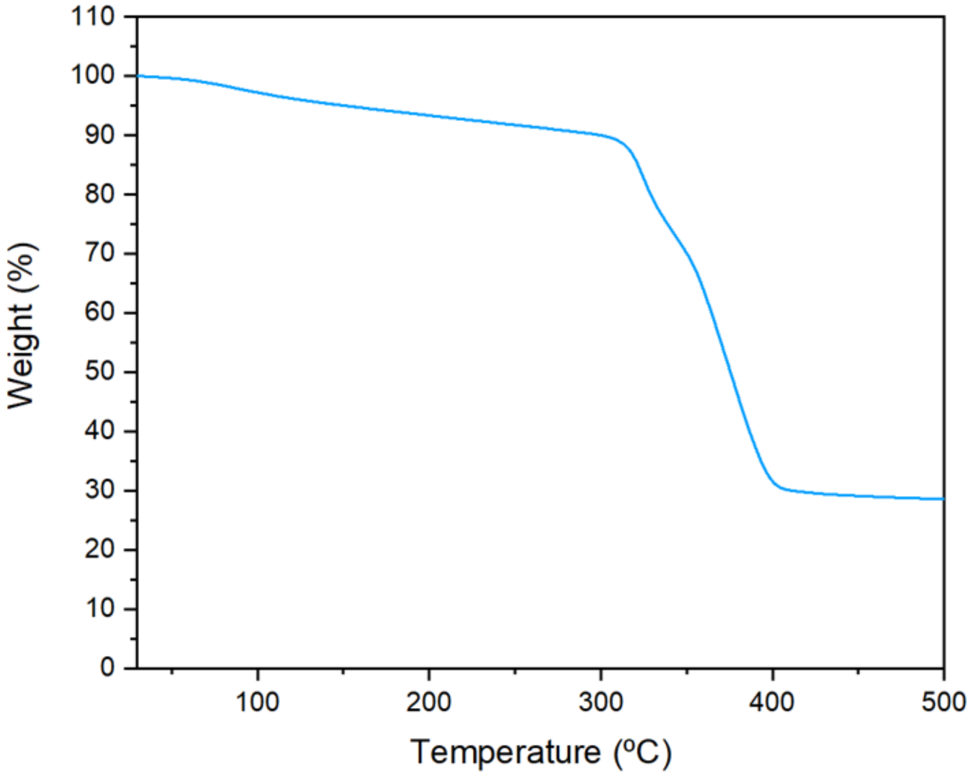


**Figure S20.** Thermogravimetric analysis of **BCN-241-MTV**. Note that the gradual weight loss until 300 ºC is attributed to free and coordinated solvent molecules. The decomposition temperature of **BCN-241-MTV** above 300 ºC agrees to reported values for Cu(II)-paddlewheel based structures.[14-15]

## Section S7. BCN-241-CHO

**Table S4.** Crystal data and structure refinement for **BCN-241-CHO**.

| | |
|---|---|
| CCDC number | 2366266 |
| Empirical formula | $C_{216}H_{96}Cu_{24}O_{144}$ |
| Formula weight | 6519.88 |
| Temperature/K | 100 |
| Crystal system | Tetragonal |
| Space group | *I*4/*m* |
| a/Å | 24.43210(10) |
| b/Å | 24.43210(10) |
| c/Å | 33.90720(10) |
| α/° | 90 |
| β/° | 90 |
| γ/° | 90 |
| Volume/Å$^3$ | 20240.14(18) |
| Z | 2 |
| $\rho_{calc}$g/cm$^3$ | 1.070 |
| μ/mm$^{-1}$ | 1.957 |
| F(000) | 6480 |
| Crystal size/mm$^3$ | 0.08 × 0.06 × 0.06 |
| Radiation/Å | Synchrotron (λ = 0.82653) |
| 2Θ range for data collection/° | 2.39 to 67.638 |
| Index ranges | $0 \leq h \leq 32$, $0 \leq k \leq 32$, $0 \leq l \leq 40$ |
| Reflections collected | 129637 |
| Independent reflections | 12029 [$R_{int}$ = 0.0415, $R_{sigma}$ = 0.0205] |
| Data/restraints/parameters | 12029/0/439 |
| Goodness-of-fit on F$^2$ | 1.083 |
| Final R indexes [I>=2σ (I)] | $R_1$ = 0.0576, w$R_2$ = 0.1911 |
| Final R indexes [all data] | $R_1$ = 0.0645, w$R_2$ = 0.1998 |
| Largest diff. peak/hole / e Å$^{-3}$ | 1.52/-0.54 |

UV-Vis analysis was used to confirm the stability of the Cu(II)-paddlewheel during the ozonolysis reaction and post work-up. To do it, 100 µL of solution before and after work-up with DMS were taken and diluted in 300 µL of methanol. Both samples were then measured at room temperature.

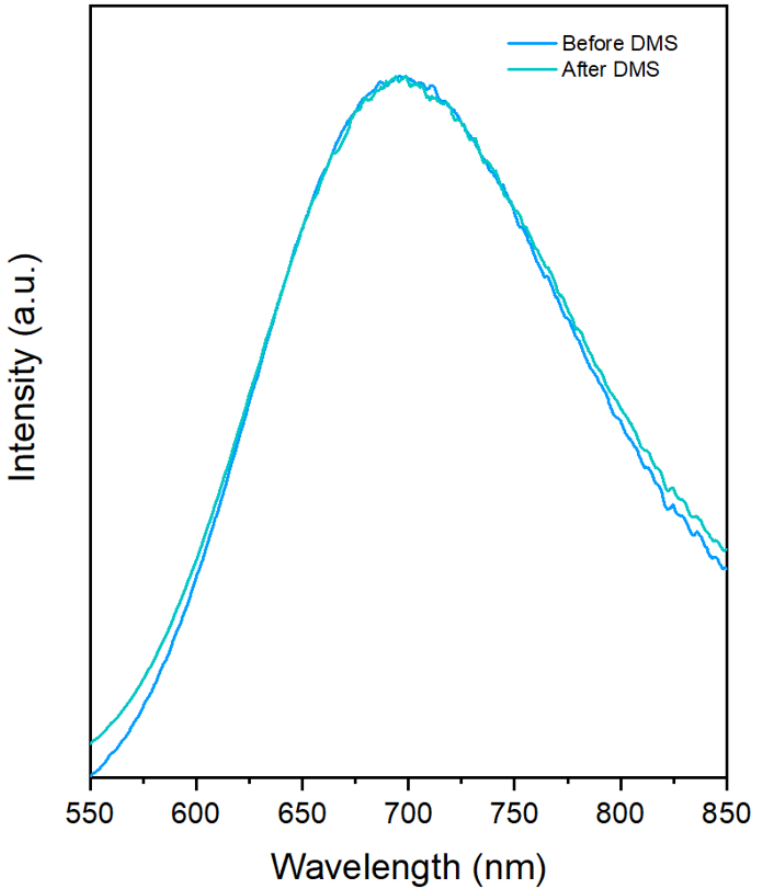


**Figure S21.** UV-Vis spectra of both supernatants before and after addition of DMS. Note that the $\lambda_{max}$ (centered at ≈ 700 nm) corresponding to Cu(II)-paddlewheel[16] was preserved after the addition of DMS, confirming its stability.

$^1$H NMR measurement of **BCN-241-CHO** was carried out by digesting 10 mg of the sample with 10 μL of DCl (20 wt. % solution in $D_2O$) in 600 μL of DMSO-$d_6$.

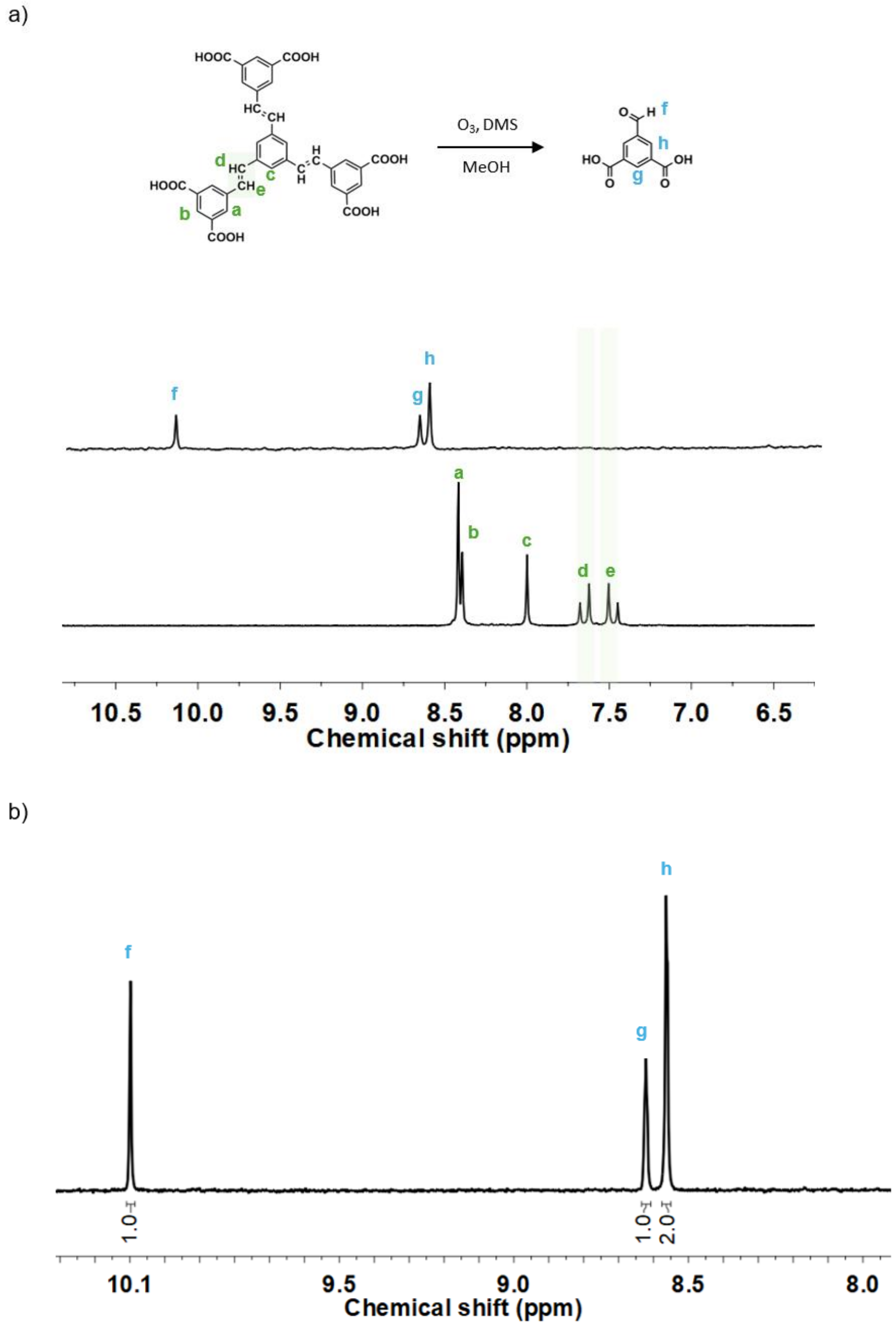


**Figure S22.** a) $^1$H NMR (300 MHz, DMSO-$d_6$/DCl) spectra of digested **BCN-241-CHO** (top) and **BCN-231** (300 MHz, DMSO-$d_6$/DCl) (bottom). Note the lack of olefinic peaks (δ= 7.66, 7.63, 7.49, 7.46 ppm, green) after ozonolysis, and b) the appearance of the signals attributed to 5-formylisophthalic acid (δ= 10.16, 8.68, and 8.62 ppm, blue).

Prior to measurement, **BCN-241-CHO** crystals were washed three times with $Et_2O$ and dissolved in DMF. Afterwards, the DMF solution was diluted (1:4) in methanol, mixed in a 1:1 ratio with a stock solution of a DCTB matrix in dichloromethane (10 mg/mL), drop-casted on the measuring plate, and dried prior to analysis. Samples were measured in positive ionization mode.

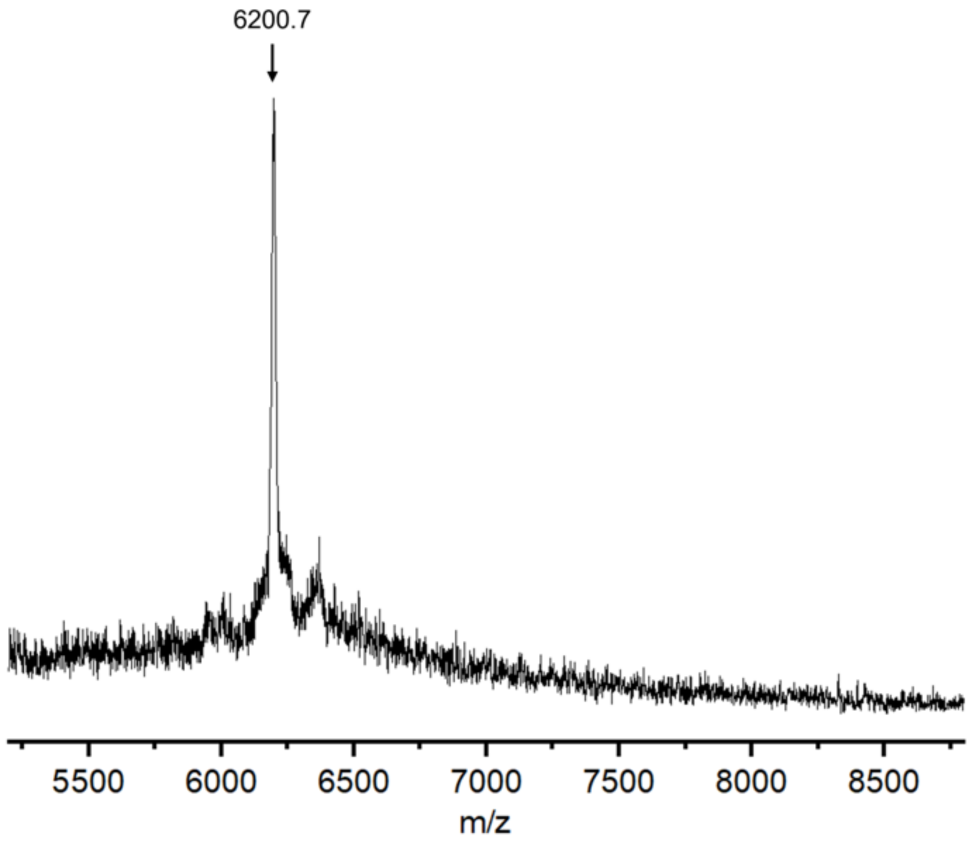


**Figure S23.** MALDI-ToF spectrum of **BCN-241-CHO** in DMF:MeOH. The sharp peak at 6200.7 matches with the expected molecular weight of $[BCN\text{-}241\text{-}CHO + H^+]^+ \cdot 2$ MeOH (m/z= 6200.6).

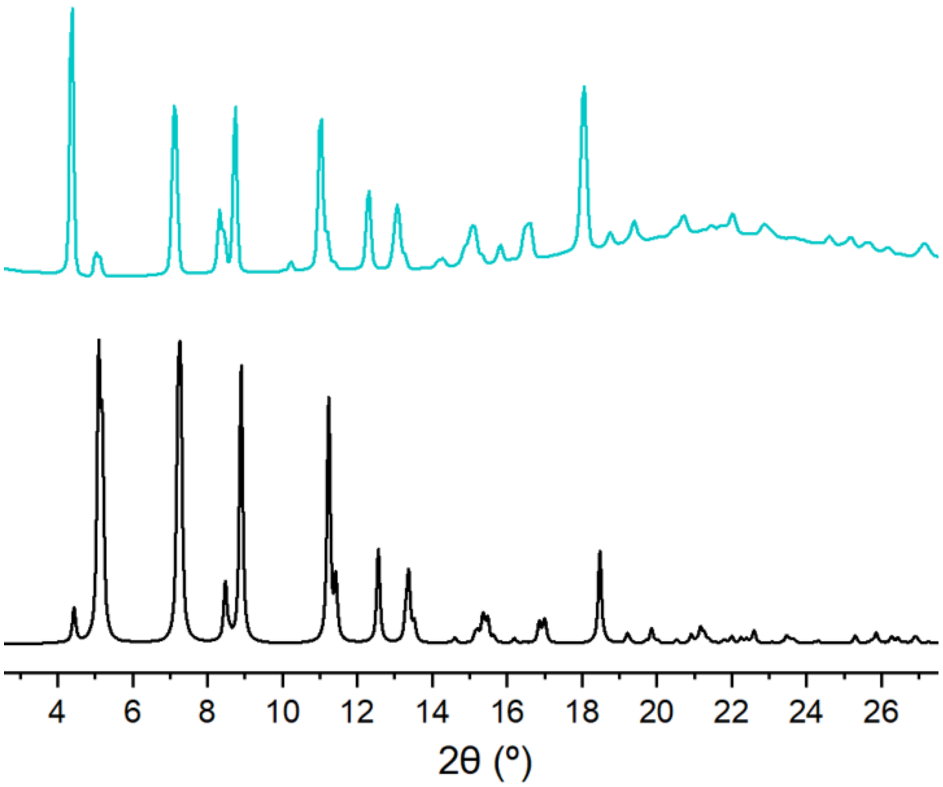


**Figure S24.** PXRD patterns of as-synthesized (turquoise) and simulated **BCN-241-CHO** (black).

Prior to this experiment, 60 mg of **BCN-241-CHO** were washed with $Et_2O$ three times. Afterwards, **BCN-241-CHO** was activated by supercritical $CO_2$ drying for 3 hours, and volumetric $N_2$ (77 K) was recorded activating the sample at 55 ºC under vacuum.

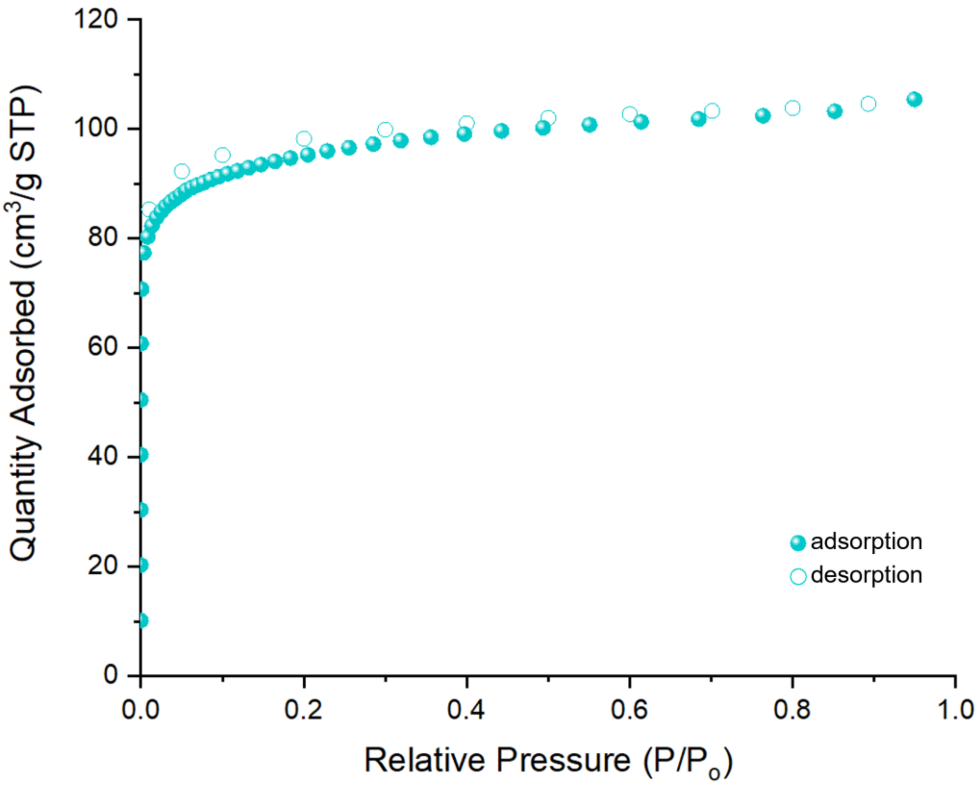


**Figure S25.** $N_2$ adsorption isotherm of **BCN-241-CHO**.

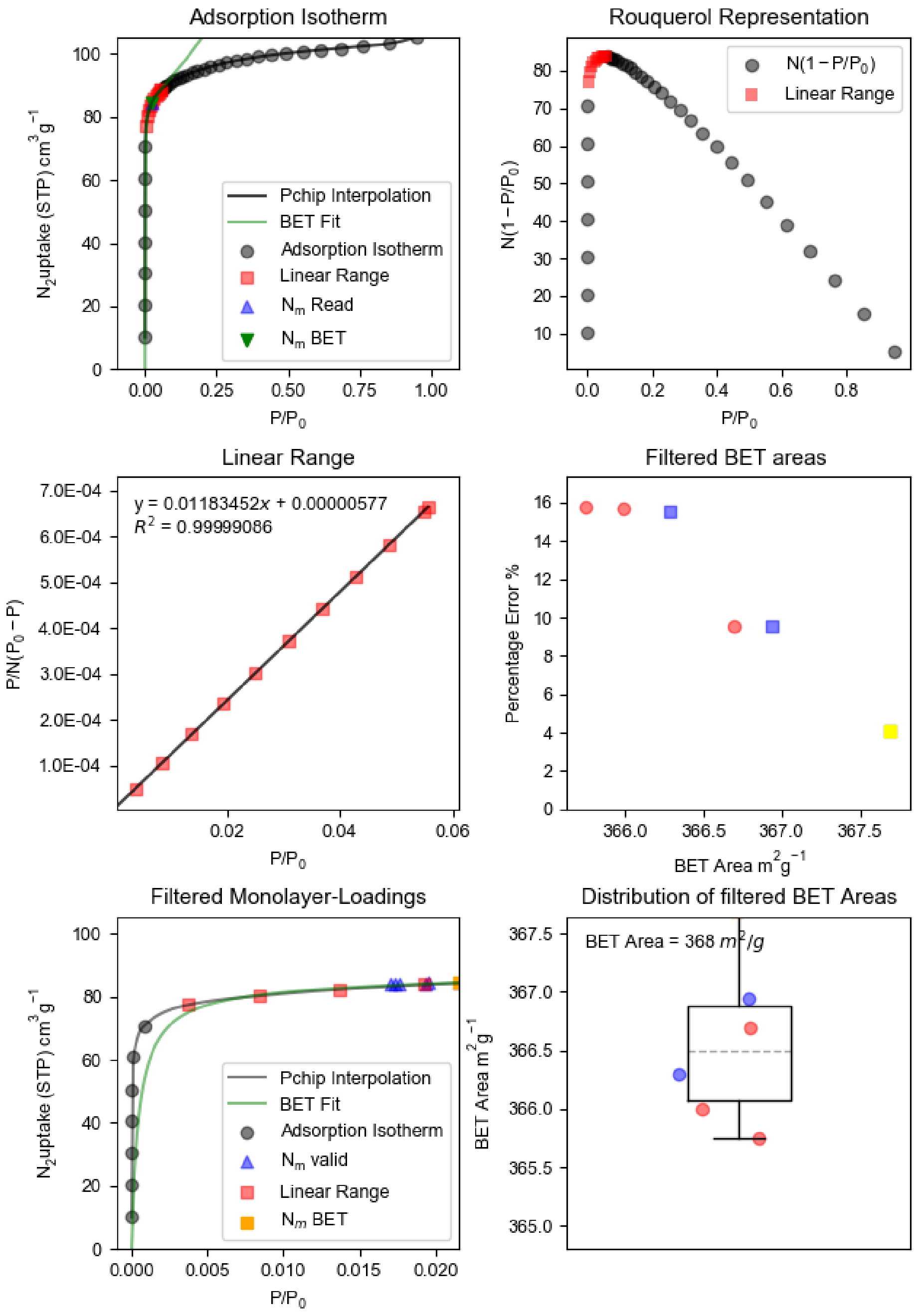


**Figure S26.** BETSI analysis of **BCN-241-CHO** ($S_{BET}$ = 368 $m^2g^{-1}$).

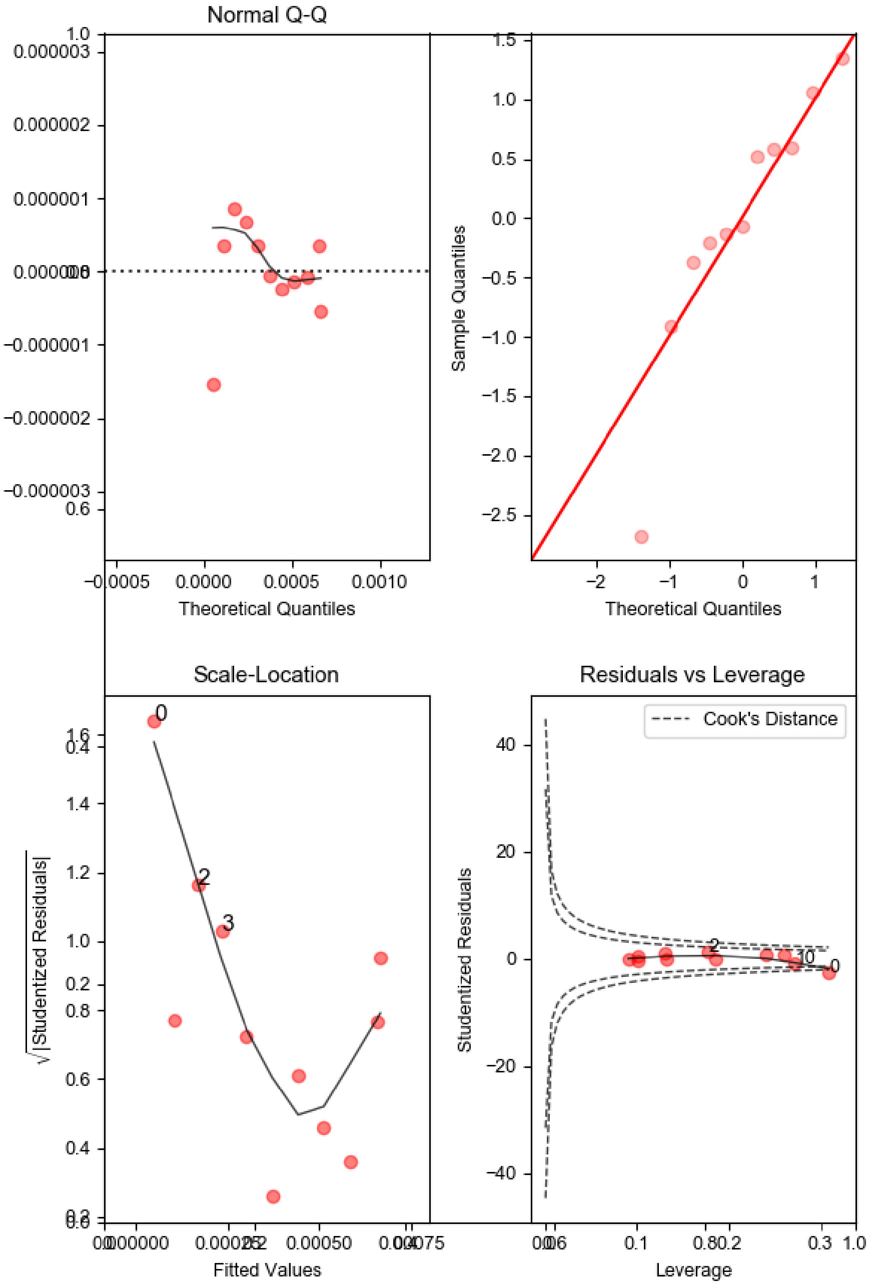


**Figure S27.** BETSI regression diagnostics of **BCN-241-CHO**.

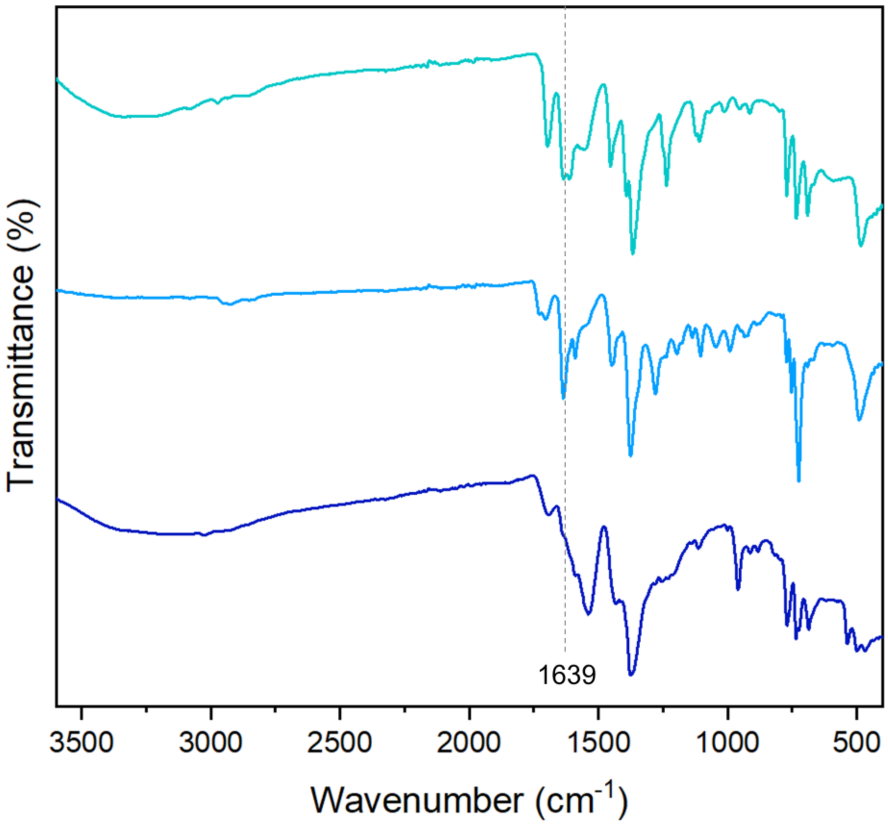


**Figure S28.** Full width of FT-IR spectrum of **BCN-241-CHO** (turquoise), **BCN-241-MTV** (cyan) and **BCN-231** (dark blue). Note that **BCN-241-MTV** and **BCN-241-CHO** exhibit an intense C=O stretch band at 1639 $cm^{-1}$ due to the cleavage of the alkene bonds of $\mathbf{H_6L_1}$ linker into carbonyl-type groups (aldehyde, ester and carboxylic acid).

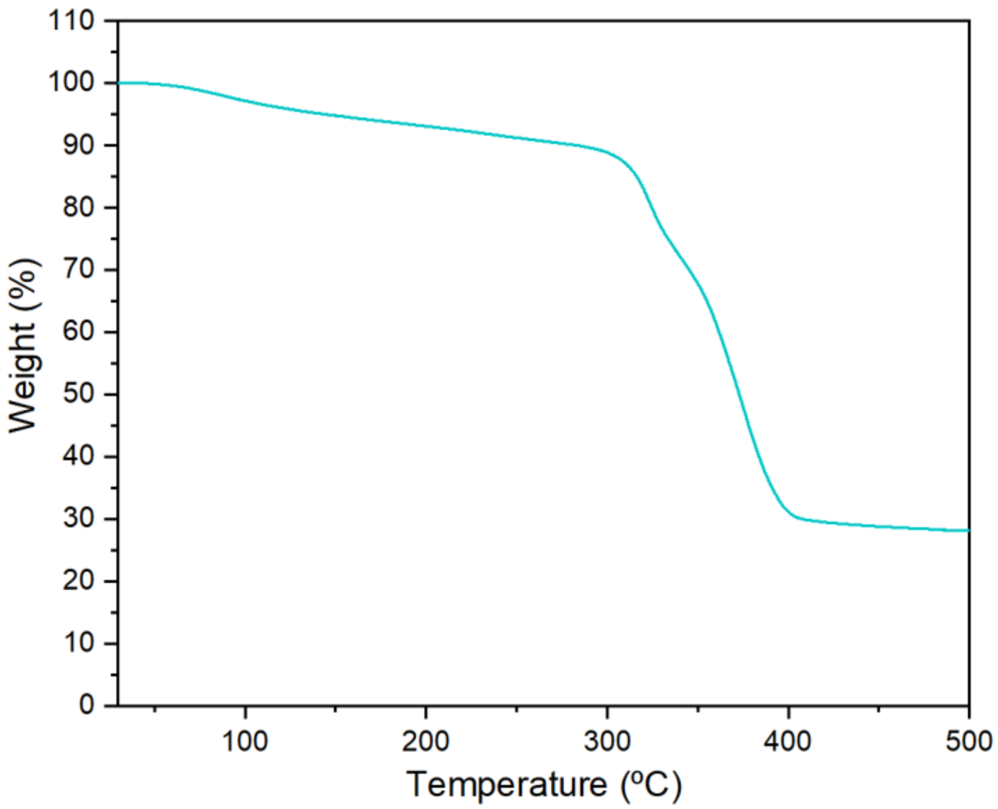


**Figure S29.** Thermogravimetric analysis of **BCN-241-CHO**. Note that the gradual weight loss until 300 ºC is attributed to free and coordinated solvent molecules. The decomposition temperature of **BCN-241-CHO** above 300 ºC agrees to reported values for Cu(II)-paddlewheel based structures.[14-15]

# References


1. Juanhuix, J.; Gil-Ortiz, F.; Cuní, G.; Colldelram, C.; Nicolás, J.; Lidón, J.; Boter, E.; Ruget, C.; Ferrer, S.; Benach, J. Developments in Optics and Performance at BL13-XALOC, the Macromolecular Crystallography Beamline at the Alba Synchrotron. *J. Synchrotron Radiat.* **2014**, *21* (4), 679–689.

2. Winter, G. *Xia2* : An Expert System for Macromolecular Crystallography Data Reduction. *J. Appl. Crystallogr.* **2010**, *43* (1), 186–190.

3. Kabsch, W. Integration, Scaling, Space-Group Assignment and Post-Refinement. *Acta Crystallogr. D Biol. Crystallogr.* **2010**, *66* (2), 133–144.

4. Evans, P. R.; Murshudov, G. N. How Good Are My Data and What Is the Resolution? *Acta Crystallogr. D Biol. Crystallogr.* **2013**, *69* (7), 1204–1214.

5. Winn, M. D.; Ballard, C. C.; Cowtan, K. D.; Dodson, E. J.; Emsley, P.; Evans, P. R.; Keegan, R. M.; Krissinel, E. B.; Leslie, A. G. W.; McCoy, A.; McNicholas, S. J.; Murshudov, G. N.; Pannu, N. S.; Potterton, E. A.; Powell, H. R.; Read, R. J.; Vagin, A.; Wilson, K. S. Overview of the *CCP* 4 Suite and Current Developments. *Acta Crystallogr. D Biol. Crystallogr.* **2011**, *67* (4), 235–242.

6. Winter, G.; Waterman, D. G.; Parkhurst, J. M.; Brewster, A. S.; Gildea, R. J.; Gerstel, M.; Fuentes-Montero, L.; Vollmar, M.; Michels-Clark, T.; Young, I. D.; Sauter, N. K.; Evans, G. DIALS: Implementation and Evaluation of a New Integration Package. *Acta Crystallogr. Sect. Struct. Biol.* **2018**, *74* (2), 85–97.

7. Evans, P. Scaling and Assessment of Data Quality. *Acta Crystallogr. D Biol. Crystallogr.* **2006**, *62* (1), 72–82.

8. Sheldrick, G. M. *SHELXT* – Integrated Space-Group and Crystal-Structure Determination. *Acta Crystallogr. Sect. Found. Adv.* **2015**, *71* (1), 3–8.

9. Farrugia, L. J. WinGX and ORTEP for Windows: An Update. *J. Appl. Crystallogr.* **2012**, *45* (4), 849–854.

10. Dolomanov, O. V.; Bourhis, L. J.; Gildea, R. J.; Howard, J. A. K.; Puschmann, H. *OLEX2* : A Complete Structure Solution, Refinement and Analysis Program. *J. Appl. Crystallogr.* **2009**, *42* (2), 339–341.

11. Spek, A. L. Single-Crystal Structure Validation with the Program PLATON. *J. Appl. Crystallogr.* **2003**, *36* (1), 7–13.

12. A P Hammersley, ESRF Internal Report, ESRF97HA02T, "FIT2D: An Introduction and Overview", **1997**.

13. Osterrieth, J.; Rampersad, J.; Madden, D. G.; Rampal, N.; Skoric, L.; Connolly, B.; Allendorf, M.; Stavila, V.; Snider, J.; Ameloot, R.; Marreiros, J.; Ania, C. O.; Azevedo, D. C. S.; Vilarrasa-García, E.; Santos, B. F.; Bu, X.-H.; Zang, X.; Bunzen, H.; Champness, N.; Griffin, S. L.; Chen, B.; Lin, R.-B.; Coasne, B.; Cohen, S. M.; Moreton, J. C.; Colon, Y. J.; Chen, L.; Clowes, R.; Coudert, F.-X.; Cui, Y.; Hou, B.; D'Alessandro, D.; Doheny, P. W.; Dinca, M.; Sun, C.; Doonan, C.; Huxley, M.; Evans, J. D.; Falcaro, P.; Riccò, R.; Farha, O. K.; Idrees, K. B.; Islamoglu, T.; Feng, P.; Yang, H.; Forgan, R.; Bara, D.; Furukawa, S.; Sanchez, E.; Gascon, J.; Telalovic, S.; Ghosha, S. K.; Mukherjee, S.; Hill, M. R.; Sadiq, M. M.; Horcajada, P.; Salcedo-Abraira, P.; Kaneko, K.; Kukobat, R.; Kenvin, J.; Keskin, S.; Kitagawa, S.; Otake, K.; Lively, R. P.; DeWitt, S. J. A.; Llewellyn, P. L.; Lotsch, B.; Emmerling, S. T.; Pütz, A.; Martí-Gastaldo, C.; Muñoz, N.; Garcia-Martinez, J.; Linares, N.; Maspoch, D.; Suarez, J. A.; Moghadam, P.; Oktavian, R.; Morris, R.; Wheatley, P.; Navarro, J.; Petit, C.; Danaci, D.; Rosseinsky, M.; Katsoulidis, A.; Schroder, M.; Han, X.; Yang, S.; Serre, C.; Mouchaham, G.; Sholl, D.; Thyagarajan, R.; Siderius, D.; Snurr, R. Q.; Goncalves, R. B.; Telfer, S. G.; Lee, S. J.; Ting, V.; Rowlandson, J.; Uemura, T.; Iiyuka, T.; Veen, M. van der; Rega, D.; Vanspeybroeck, V.; Lamaire, A.; Rogge, S.; Walton, K.; Bingel, L. W.; Wuttke, S.; Andreo, J.; Yaghi, O.; Zhang, B.; Yavuz, C.; Nguyen, T.; Zamora, F.; Montoro, C.; Zhou, H.-C.; Angelo, K.; Fairen-Jimenez, D. How Reproducible Are Surface Areas Calculated from the BET Equation? *Adv. Mater.* **2022**, 2201502.

14. Chen, B., Eddaoudi, M., Hyde, S. T., O'Keeffe, M., & Yaghi, O. M. Interwoven Metal-Organic Framework on a Periodic Minimal Surface with Extra-Large Pores. *Science* **2001**, *291* (5506), 1021-1023.

15. Yan, Y., Lin, X., Yang, S., Blake, A. J., Dailly, A., Champness, N. R., Hubberstey, P., & Schröder, M. Exceptionally high $H_2$ storage by a metal–organic polyhedral framework. *Chem. Commun.* **2009**, *9*, 1025 -1027.

16. Selvakumar, P. M.; Nadella, S.; Sahoo, J.; Suresh, E.; Subramanian, P. S. Copper(II) Bis-Chelate Paddle Wheel Complex and Its Bipyridine/ Phenanthroline Adducts. *J. Coord. Chem.* **2013**, *66* (2), 287–299.
